# Augmenting drug–carrier compatibility improves tumour nanotherapy efficacy

**DOI:** 10.1038/ncomms11221

**Published:** 2016-04-13

**Authors:** Yiming Zhao, François Fay, Sjoerd Hak, Jose Manuel Perez-Aguilar, Brenda L. Sanchez-Gaytan, Brandon Goode, Raphaël Duivenvoorden, Catharina de Lange Davies, Astrid Bjørkøy, Harel Weinstein, Zahi A. Fayad, Carlos Pérez-Medina, Willem J. M. Mulder

**Affiliations:** 1 Translational and Molecular Imaging Institute, Icahn School of Medicine at Mount Sinai, New York, New York 10029, USA; 2 Department of Circulation and Medical Imaging, The Norwegian University of Science and Technology, 7030 Trondheim, Norway; 3 Department of Physiology and Biophysics, Weill Cornell Medical College of Cornell University, New York, New York 10065, USA; 4 IBM Thomas J. Watson Research Center, Yorktown Heights, New York 10598, USA; 5 Department of Vascular Medicine, Academic Medical Center, Amsterdam 1105 AZ, The Netherlands; 6 Department of Physics, The Norwegian University of Science and Technology, 7030 Trondheim, Norway; 7 The HRH Prince Alwaleed Bin Talal Bin Abdulaziz Alsaud Institute for Computational Biomedicine, Weill Cornell Medical College of Cornell University, New York, New York 10065, USA; 8 Department of Medical Biochemistry, Academic Medical Center, 1105 AZ Amsterdam, The Netherlands

## Abstract

A major goal of cancer nanotherapy is to use nanoparticles as carriers for targeted delivery of anti-tumour agents. The drug–carrier association after intravenous administration is essential for efficient drug delivery to the tumour. However, a large number of currently available nanocarriers are self-assembled nanoparticles whose drug-loading stability is critically affected by the *in vivo* environment. Here we used *in vivo* FRET imaging to systematically investigate how drug–carrier compatibility affects drug release in a tumour mouse model. We found the drug's hydrophobicity and miscibility with the nanoparticles are two independent key parameters that determine its accumulation in the tumour. Next, we applied these findings to improve chemotherapeutic delivery by augmenting the parent drug's compatibility; as a result, we achieved better antitumour efficacy. Our results help elucidate nanomedicines' *in vivo* fate and provide guidelines for efficient drug delivery.

## Abstract (web summary)

The *in vivo* anticancer efficacy of nanoparticle-mediated drug delivery depends on the association between the drug and its carrier. Here, the authors use FRET to show that the drug hydrophobicity, and miscibility with the carrier, influence nanoparticle accumulation in murine tumour models.

## Graphical abstract

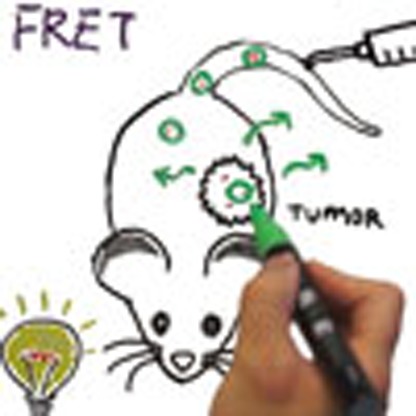

Nanoparticle therapy is becoming increasingly relevant in cancer treatment^1^,^2^,^3^,^4^,^5^,^6^,^7^. The first clinical nanoparticle chemotherapy formulation was approved 20 years ago^8^, and preclinical nanomedicine has undergone unprecedented growth in the past 10 years^1^,^4^. The majority of nanotherapies use nanoparticles as carriers to improve the parent drug's aqueous dispersity, bioavailability, toxicity profile and pharmacokinetic properties^2^. Moreover, surface functionalization allows specific interactions with target cells^5^.

Self-assembled nanoparticles, such as polymeric micelles^6^,^9^,^10^,^11^, lipid nanoparticles^12^ or nanoemulsions^13^,^14^, are widely used as delivery vehicles for poorly water-soluble compounds. There is particular interest in and increasingly study of second-generation self-assembled polymeric nanoparticle platforms that allow controlled drug release, and some of these have entered clinical trials^15^,^16^. Such platforms typically carry chemotherapeutic agents that are physically entrapped in or attached to particles' hydrophobic cores^17^,^18^. However, the majority of these self-assembled structures have problems with drug-loading stability, which is strongly influenced by the *in vivo* environment^19^,^20^,^21^,^22^, in that blood components can act as competing drug acceptors^23^. Interactions between polymeric nanoparticles and blood components have been reported to cause drug leakage^24^. Therefore, thoroughly understanding *in vivo* drug–carrier association stability and dissociation kinetics should improve delivery efficiency and, as a result, therapeutic efficacy.

The drug's hydrophobicity^19^ and its miscibility with the polymeric matrix^25^,^26^ were known to determine nanoparticle drug loading. However, it remains unclear how these properties contribute to the drug–carrier association in circulation, subsequent tumour delivery efficiency and resulting therapeutic efficacy. Systematically investigating the effect of drug hydrophobicity and miscibility *in vivo* is therefore an imperative step towards improving nanoparticle therapeutics.

Most studies typically determine drug release *in vitro* but seldom achieve crucial *in vivo* characterization due to technical challenges. To address this knowledge gap we built a dual fluorescently labelled nanoparticle that allowed us to monitor the drug–carrier association using Förster resonance energy transfer (FRET)^27^,^28^. Through rational derivatization, we were able to fine-tune a model drug's hydrophobicity and miscibility. In addition, we used *in vivo* optical imaging studies on a breast cancer mouse model to identify key parameters that determine drug–carrier compatibility. Our results show that augmenting drug–carrier compatibility significantly improves tumour accumulation. These findings can serve as drug delivery efficiency guidelines that can be applied to widely used chemotherapies, such as doxorubicin, to improve their antitumour efficacy.

## Results

### Studying nanoparticle drug release using FRET

**Figure 1:**
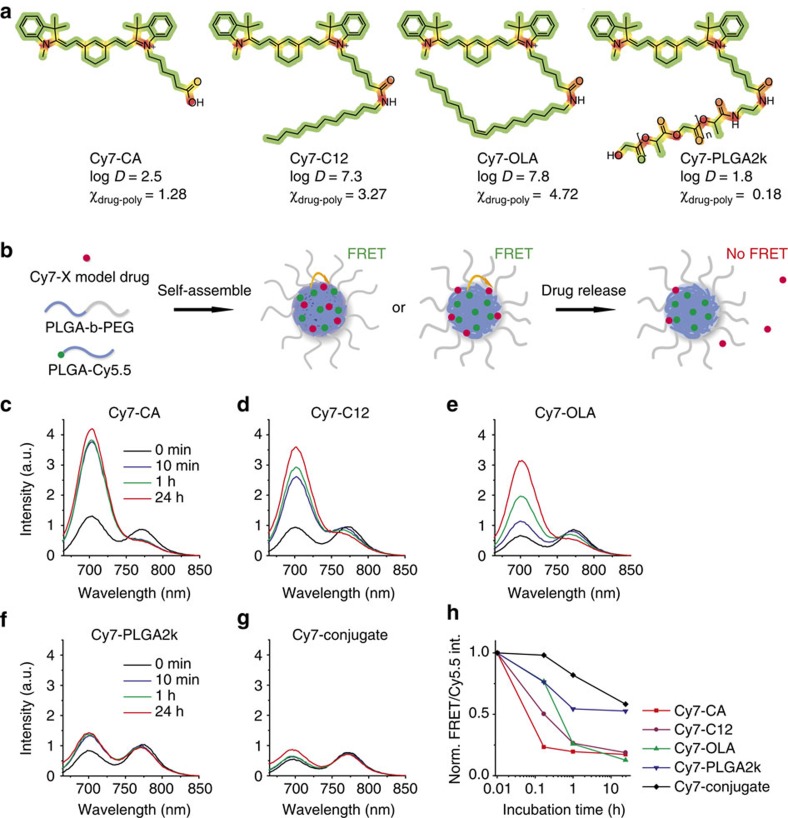
Cy7-X model drugs release in serum from Cy5.5-NP:Cy7-X FRET nanoparticles. (**a**) Chemical structures of Cy7-X (X=CA, C12, OLA and PLGA2k) with hydrophobic segments presented in green and hydrophilic ones in red. The Cy7-X molecule's overall hydrophobicity (log *D*) and miscibility (*χ*_drug-poly_) in PLGA matrix were modified by varying the tail part X. (**b**) Schematic showing Cy5.5-NP:Cy7-X FRET nanoparticle formed through self-assembly of PLGA(blue)–PEG(grey) block copolymer and Cy5.5(green)-conjugated PLGA in the presence of Cy7-X (red). FRET is achieved when Cy7-X associated with particle and reduced when Cy7-X is released. (**c**–**g**) Emission spectra of Cy5.5-NP:Cy7-X and Cy5.5-Cy7-NP incubated with FBS at 37 °C after indicated times. The significant differences in release rates observed for different model drugs are displayed in **h**. int., intensity; Norm., normalized.

We first created a range of poorly water-soluble model drugs with different physicochemical properties (Fig. 1a). These model drugs consisted of a near-infrared fluorescent (NIRF) dye, Cy7, with varying tail components X (X=carboxylic acid (CA), C12, OLA and PLGA2k). The tails modify the overall Cy7-X molecule's hydrophobicity and miscibility for matrix polymers (Supplementary Table 1). Increasing alkyl chain length from Cy7-CA to Cy7-C12 to Cy7-OLA raises hydrophobicity (represented by distribution coefficients, log *D* at pH=7.4)^29^, while conjugation to a short oligomer chain (Cy7-PLGA2k) improves miscibility, in the context of this study, with poly(D,L-lactide-co-glycolide) (PLGA) (represented by Flory–Huggins interaction parameters, *χ*_drug-poly_)^30^. It has to be stressed that although related, hydrophobicity and miscibility are two independent parameters, which means a drug with good miscibility may not necessarily have a high hydrophobicity (for example, Cy7-PLGA2k) and vice versa (for example, Cy7-OLA). The nanoparticles were subsequently produced through self-assembly of the block-copolymer PLGA–block-poly(ethylene glycol) (PLGA–PEG) and Cy5.5-conjugated high-molecular-weight PLGA as the core material. Cy7-X model drugs were incorporated during nanoparticle formation (Fig. 1b, see nanoparticles' characterizations in Supplementary Table 2)^6^. In these systems, Cy5.5 in the core serves as a FRET donor, while Cy7-X acts as a FRET acceptor when associated with the nanoparticle. Using a fixed Cy5.5 core content, we observed that raising the Cy7-X nanoparticle loading increases FRET emission (peak at 770 nm) and correspondingly decreases Cy5.5 emission (at 700 nm). In addition, we found a linear relation between the average Cy7-X loading per particle and measured FRET/Cy5.5 intensity ratio (FRET ratio) (Supplementary Fig. 1)^31^. On the basis of this, we were able to estimate the amount of Cy7-X on the particle through direct emission spectra measurements.

The synthesized nanoparticles, hereafter referred to as Cy5.5-NP:Cy7-X, were stable in phosphate-buffered saline (PBS), and their drug release half-life was generally over 24 h at 37 °C (Supplementary Fig. 2). However, incubations with fetal bovine serum (FBS) showed markedly different results. We observed marked emission spectra changes that indicate much faster Cy7-X releases in FBS. Figure 1c–g present the emission spectra recorded for four types of Cy5.5-NP:Cy7-X and the control particle (both Cy5.5 and Cy7 were conjugated to the PLGA core), before and after incubation with FBS at 37 °C for 10 min, 1 h and 24 h. The summarized data in Fig. 1h show clear Cy7-X release rate differences: the quickest is Cy7-CA, which releases within a few minutes, whereas Cy7-PLGA2k release takes several hours. The control particles' relatively unchanged spectra indicate that the PLGA–PEG nanoparticles themselves did not disintegrate during incubation.

### Drug properties determine release rate from nanoparticles

**Figure 2:**
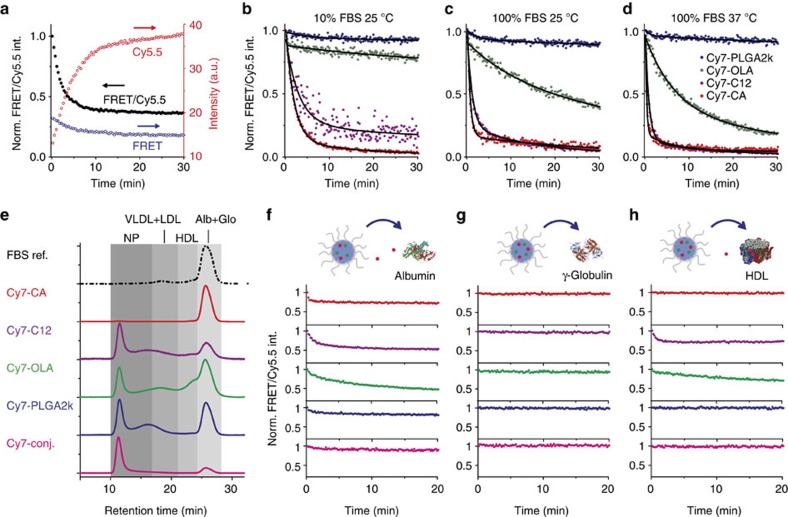
*In vitro* drug release dynamics of Cy5.5-NP:Cy7-X in serum. (**a**) A typical measurement of release dynamics, determined by recording time-dependent fluorescence in Cy5.5 channel (red circles, *λ*_Exc_=620 nm and *λ*_Emi_=700 nm) and FRET channel (blue circles, *λ*_Exc_=620 nm and *λ*_Emi_=780 nm). The normalized (Norm.) FRET/Cy5.5 intensity (int.) ratio (black solid dots) represents the amount of Cy7-X still associated with nanoparticles. Arrows indicate the corresponding *y* axes. (**b**–**d**) FBS concentration (**b**,**c**) and temperature (**c**,**d**) affects release rate. Data are fitted with a two-compartment decay model (black curves). (**e**) FPLC analysis of Cy7-X distribution in incubation mixtures of FBS and Cy5.5-NP:Cy7-X. Chromatograms of the FBS reference (ref.) and Cy7-X were recorded through absorbance at 250 and 760 nm, respectively. (**f**–**h**) Release dynamics of Cy5.5-NP:Cy7-X mixed with selected single-plasma protein solutions: albumin (**f**); γ-globulin (**g**); and high-density lipoprotein (HDL) (**h**).

To study the *in vitro* release dynamics of Cy7-X in FBS, we performed time-dependent fluorescence measurements^31^. In a typical dynamic experiment, as shown in Fig. 2a, mixing Cy5.5-NP:Cy7-X and FBS results in gradually increased Cy5.5 intensity and simultaneously decreased FRET intensity, in which the FRET ratio is a drug release measure. We investigated environmental factors affecting the Cy7-X release rate by conducting dynamic experiments at different FBS concentrations and varying temperatures. The FRET ratio decay curves for representative measurements are plotted in Fig. 2b–d. Extensive dynamic studies on concentration and temperature effects can be found in Supplementary Fig. 3, while decay curve fitting results are listed in Supplementary Table 3. We found that the different Cy7-Xs release more quickly at increased FBS concentrations and at higher temperatures. The release rates also depend on the Cy7-X's hydrophobicity and miscibility, following the order: CA>C12>OLA>PLGA2k.

We used fast protein liquid chromatography (FPLC) to separate and analyse the particle-serum mixtures after 2 h of incubation (Fig. 2e)^32^. We observed Cy7-X migrating from the original carrier particles to different serum components, with distributions varying among the albumin and globulin portion, high-density lipoprotein and low-density lipoprotein^28^. These interactions were confirmed by dynamic experiments with single-plasma protein solutions (Fig. 2f–h). Interestingly, Cy5.5-NP:Cy7-X in albumin solutions and in FBS showed very similar drug release curves, possibly because albumin is the dominating drug carrier in the serum. We performed additional dynamic studies to further explore the universality of this drug exchange phenomenon, in which we subsequently observed Cy7-X's inter-particle, as well as inter-albumin, and albumin-to-particle exchange, all with similar dynamic characteristics (Supplementary Fig. 4). Moreover, we also performed experiments in human serum and observed the drug release dynamics to depend on serum protein composition and concentration (Supplementary Fig. 5). This implies that the nanoparticle drug–carrier association features need to be carefully considered when translating nanomedicines to the clinic.

### Molecular simulations disclose drug-carrier interactions

**Figure 3:**
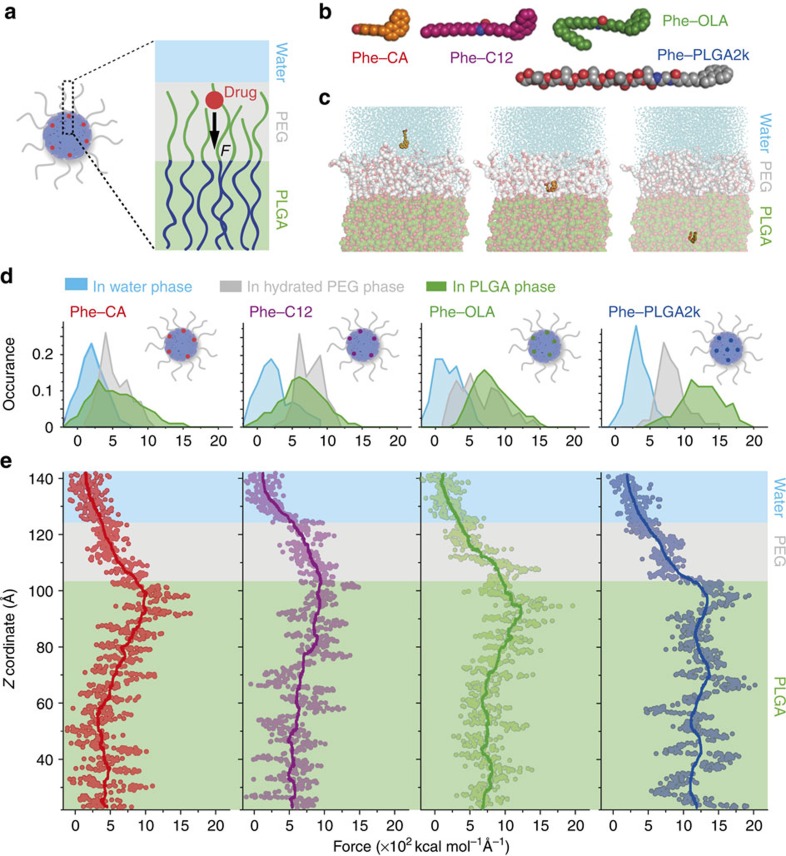
Computer simulations on drug–nanoparticle interactions and predictions of drug-loading positions. (**a**) Schematic illustration of the SMD simulations. A three-phase model system was constructed to simulate the environment of the drug molecules in a colloidal PLGA–PEG micelle. From the top to bottom the phases are as follows: water (blue); hydrated PEG (grey); and PLGA (green). Different Phe-X (X=CA, C12, OLA and PLGA2k) model drugs (red dot) were steered by a force *F* (black arrow) progressing along the *Z*-coordinate to visit all three phases. (**b**) The molecular structures (in space-filling renderings) of the four Cy7-X analogues. (**c**) Snapshots of the simulated systems. The Phe-CA compound (orange) is depicted as it moves along the water (left), the PEG (middle) and the PLGA (right) phase. Oxygen atoms (red), PLGA carbons (green), PEG carbon (grey) and water oxygen atoms (cyan). (**d**,**e**) Results from the SMD simulations. The forces applied on the Phe-X along the designed pathway (*Z*-coordinate) are plotted against the position (**e**), with the different phases indicated by the same colour scheme as in **a**. The solid lines, meant to guide the eye, are obtained through adjacent-averaging method. The distributions of the force values in each phase are displayed on top of the panels using the same colour code (**d**). Possible loading positions for each drug model are indicated in the nanoparticle schematics in **d**.

To shed light on the above observations, we used all-atom steered molecular dynamics (SMD) computer simulations^33^ to help understand how drugs' properties—most notably those determining their location—affect their interactions with the nanoparticles (Fig. 3)^34^. We first constructed a three-phase model system to simulate the environment of drug molecules in a colloidal PLGA–PEG nanoparticle. It consisted of a PLGA phase, representing the core of the nanoparticle, a PEG phase, representing the nanoparticle's hydrated PEG corona, and a water phase, representing the bulk aqueous environment (Fig. 3a). Next, we constructed four drug models with different hydrophobicity and PLGA matrix miscibility. To simplify the simulations, we used a generic drug-like structure consisting of a phenanthrene (Phe) moiety, which was attached to the corresponding tail component (that is, -CA, -C12, -OLA or -PLGA2k) to modulate physicochemical properties that resemble the Cy7-X model drugs (Fig. 3b).

Molecular interactions between the Phe-X molecules and the environment at different positions relative to the nanoparticle were investigated by applying an external probing force (*F*). The four different Phe-Xs were moved along a pathway (*Z*-direction) designed to explore each phase sequentially at a constant velocity (Fig. 3a,c). Higher *F* values indicate higher local drug–environment interactions and lower local free energies^35^. Therefore, the SMD *F* trajectories provide inferential information about the favoured loading positions for each Phe-X compound. Generally, we observed stronger forces in the PLGA phase than in both the water or PEG phases, consistent with Phe-X's nanoparticle-loading ability (Fig. 3d). The force distribution differentials between the PLGA and water phases indicate how strong a drug molecule is associated with the nanoparticle. The SMD results predict the fast-to-slow ranking (Phe-CA>Phe-C12>Phe-OLA>Phe-PLGA2k) in line with the experimental results for the Cy7-X compounds (Fig. 3d). Interestingly, for Phe-PLGA2k, the *F* values remain high throughout the entire PLGA phase, suggesting a homogenous dispersion in the nanoparticle core. On the other hand, for Phe-CA, Phe-C12 and Phe-OLA, the *F* values reached a maximum at the PEG to PLGA interface, which decreased as the compounds moved into the PLGA phase. This implies that these compounds are less likely to be incorporated in the PLGA core and prefer to stay within the PEG–PLGA interface (Fig. 3e).

The SMD data, in conjunction with the dynamic *in vitro* experiments, provide valuable information on the relationship between drug–carrier compatibility and the release rate. In a specific system, both hydrophobicity and miscibility contribute to the drug–carrier association and hence affect the drug release, but they are based on very different mechanisms. Drugs with a high polymer matrix miscibility, such as Cy7-PLGA2k, have a homogenous core distribution, display better nanoparticle association and are less affected by environmental conditions. On the other hand, low-miscible drugs, such as Cy7-CA, Cy7-C12 and Cy7-OLA, have weaker drug–carrier interactions and mostly localize at the interface between the PLGA core and the hydrated PEG corona (as illustrated in Figs 1b and 3d)^36^. At these interfaces, the drugs are stabilized mainly through hydrophobic interactions, and hence they can dissociate from the nanoparticles due to thermal fluctuations^37^. Therefore, when stored in PBS, in the absence of alternative receptors, the surface-attached drug molecules merely exchange between the nanoparticles (Supplementary Fig. 4a,b). Under these conditions, the entire system is at dynamic equilibrium without observable net drug release. However, if additional competing drug acceptors, for example, serum proteins, are available, drug molecules can migrate to these serum components^38^. The drug exchange rate is controlled by temperature, carrier concentration and dissociation activation energies that are proportional to the drugs' hydrophobicity^39^. A detailed discussion on the drug exchange mechanism can be found in Supplementary Discussion.

### Intravital microscopy shows premature drug release

**Figure 4:**
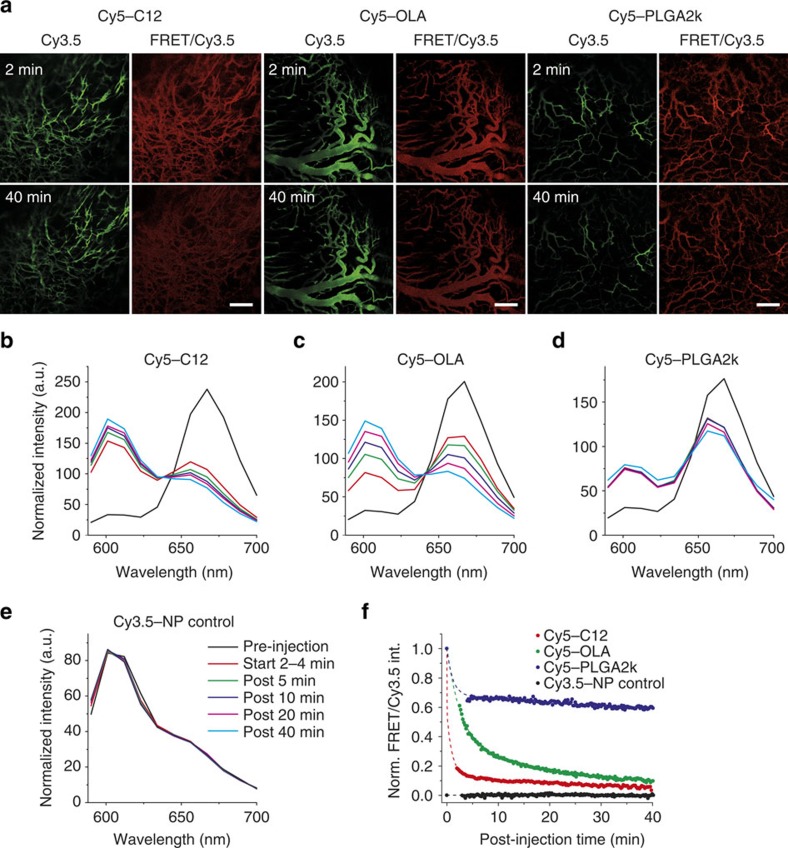
Intravital microscopy investigation of drug release dynamics in the vasculature. Window chamber mice were injected intravenously with Cy3.5-NP:Cy5-X (X=C12, OLA and PLGA2k) FRET and Cy3.5-NP non-FRET control nanoparticles and continuously observed for 1 h. (**a**) Representative images of Cy3.5 channel (green) and FRET/Cy3.5 ratio (red) at 2 and 40 min post injection. Scale bars, 100 μm. (**b**–**e**) Emission spectra of Cy3.5-NP/Cy5-X in the vasculature, at selected post-injection times. The legend in **e** applies to **b**–**e**. (**f**) The drug release dynamics in circulation were measured via spectral imaging. The FRET/Cy3.5 intensity ratio is normalized to the value before injection. Dotted lines are for vision guidance.

*In vivo* drug release dynamics in the vasculature were investigated in real time using intravital confocal laser scanning microscopy on a window chamber mouse model^40^. To accommodate the microscope settings, we synthesized Cy3.5-NP:Cy5-X (X=C12, OLA and PLGA2k) with properties similar to Cy5.5-NP:Cy7-X (see Cy5-Xs' structures and properties in Supplementary Fig. 6 and Supplementary Table 1, respectively). After we intravenously administered Cy3.5-NP:Cy5-X FRET nanoparticles, we recorded time-dependent confocal images using different channels. See selected images in Fig. 4a. In these experiments the FRET ratio imaging is concentration-independent. We observed that Cy5-C12 had the fastest FRET ratio reduction. Next, we acquired in-depth quantitative information via spectral imaging. In Fig. 4b–d, the blood vessel spectra for Cy3.5:Cy5-X and non-FRET control are plotted at selected post-injection time points. The spectra shape's gradual evolution provides a direct impression of drug release in circulation. Figure 4f summarizes time-dependent FRET ratios for different model drugs. All three particles demonstrated a burst release in the first few minutes, but to very different extents: Cy5-PLGA2k and Cy5-OLA remained associated with the particle much more strongly than Cy5-C12. At later time points, the dissociation rates gradually decreased and followed the order: C12>OLA>PLGA2k. Remarkably, although the nanoparticle–environment interactions *in vivo* are much more complicated than those that occur during *in vitro* experiments, we nevertheless observed the same trend on release rates. These results confirmed that either better miscibility or higher hydrophobicity slows drug release in circulation.

### NIRF imaging shows differential drug release in tumours

**Figure 5:**
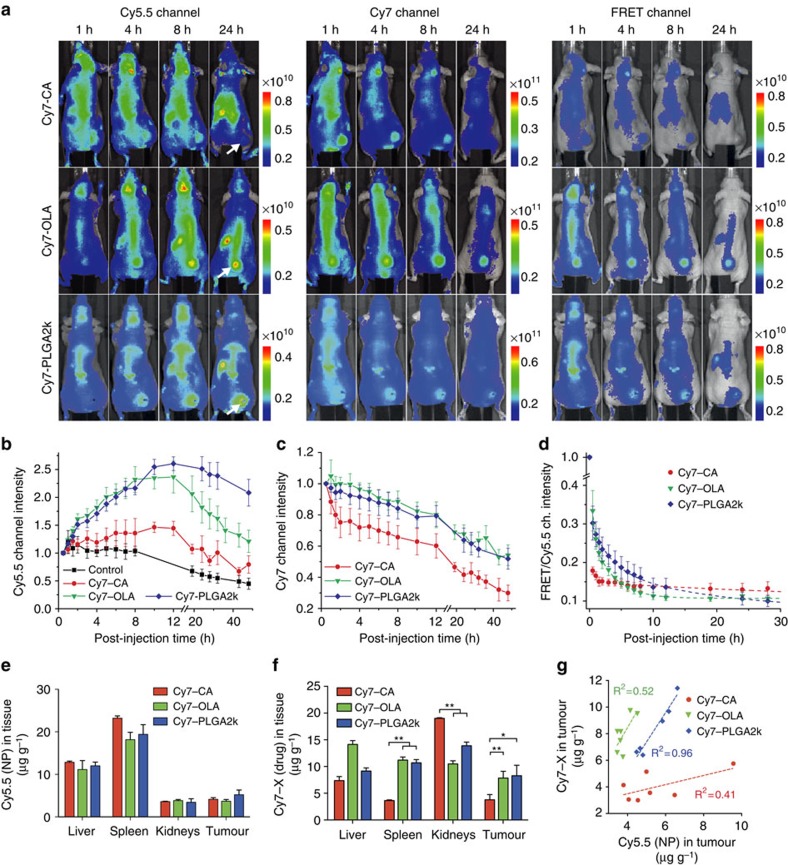
Accumulation and subsequent drug release of Cy5.5-NP:Cy7-X in tumour. (**a**) Representative NIRF images obtained at selected post-injection times in three optical channels: Cy5.5 (*λ*_Exc_=640 nm and *λ*_Em_=720 nm); Cy7 (*λ*_Exc_=745 nm and *λ*_Em_=800 nm); and FRET (*λ*_Exc_=640 nm and *λ*_Em_=800 nm). The right flank tumours are indicated with arrows. The units are radiant efficiency (p s^−1^ cm^−2^ sr^−1^)/(μW cm^−2^). (**b**–**d**) The mean intensities from the tumour area (*n*=8 mice per group) in the Cy5.5 channel (**b**), the Cy7 channel (**c**) and FRET/Cy5.5 intensity ratios (**d**) are plotted against post-injection time. (**e**,**f**) The biodistribution (*n*=3) of Cy5.5-NP/Cy7-X in tumour and major organs at 24 h post injection, as evaluated by Cy5.5 tissue concentration (representing Cy5.5-NP carrier particles concentration) (**e**) and Cy7 tissue concentration (representing Cy7-X model drug concentration) (**f**). Data are shown as mean±s.d. *P<0.01, **P<0.001 using a Mann–Whitney test. (**g**) Correlations between Cy5.5 and Cy7 concentrations in tumour tissues samples (*n*=3) at 24 h post injection. Dotted lines are the linear fits with *R*^2^ values given in the plot.

To investigate tumour accumulation and subsequent drug release in a murine tumour model *in vivo*, we used NIRF imaging. Four groups of nude mice bearing MDA-MB-231 tumour xenografts on their right flanks (*n*=8 per group) were intravenously injected with Cy5.5-NP:Cy7-X (X=CA, OLA and PLGA2k) FRET or Cy5.5-NP non-FRET control nanoparticles. We determined the circulation half-lives of Cy5.5-NP carriers and loaded Cy7-Xs (Supplementary Fig. 7), and we recorded NIRF images using the Cy5.5, Cy7 and FRET channels at several time points up to 48 h (Fig. 5a). Tumour accumulation could be observed as early as 30 min post injection, and distinctive, temporal signal intensities subsequently evolved for the different nanoparticles. Average intensities from tumour areas are summarized in Fig. 5b–d, which also provide a quantitative group comparison. In the Cy5.5 channel (Fig. 5b), the control nanoparticle group shows accumulation and retention kinetics without the FRET effect. In the groups injected with the FRET nanoparticles, the signal represents the combinatory effect of nanoparticle accumulation and Cy5.5 de-quenching as a result of drug (Cy7-Xs) dissociation. We observed the Cy5.5-NP:Cy7-OLA and Cy5.5-NP:Cy7-PLGA signal increases were significantly higher and longer lasting than Cy5.5-NP:Cy7-CA. The Cy7 channel reports on Cy7-X retention in the tumour (Fig. 5c). In line with results in the Cy5.5 channel, Cy5.5-NP:Cy7-OLA and Cy5.5-NP:Cy7-PLGA better retain the model drugs. In the first 1–2 h post injection, the fluorescence intensity from the tumour area originates from both circulating and accumulated nanoparticles. In this timespan, the gradually increasing tumour accumulation signal becomes appreciable when nanoparticles are clearing from circulation. Finally, the decay of FRET ratios represents a direct drug release measure in the tumour area. Figure 5d shows a higher FRET ratio for Cy7-OLA and Cy7-PLGA2k at 30 min post injection, a result that indicates they were still associated with the particle to a greater extent than Cy7-CA, which had mostly already dissociated. These findings accord with the intravital microscopy results. Moreover, we found the drug release rate in the tumour followed a similar order as in circulation (PLGA2k>OLA>CA), albeit at a slower rate.

Furthermore, biodistribution studies of Cy5.5-NP:Cy7-X (X=CA, OLA and PLGA2k) 24 h after administration showed that while all the Cy5.5-NP carrier nanoparticles displayed very similar distribution patterns in major organs and tumours (Fig. 5e), the distribution of the different Cy7-Xs greatly varies (Fig. 5f). In the tumour, Cy7-OLA's and Cy7-PLGA2k's tissue concentrations were significantly higher than Cy7-CA's. This implies that the ‘stickier' the drug is to the particle, the higher the tumour uptake will be. In contrast, Cy7-CA had the highest kidney association, which suggests drug release in the blood and subsequent renal accumulation and clearance. *Ex vivo* analyses of tumour tissue samples (Fig. 5g) also revealed that the nanoparticle carriers retain more Cy7-PLGA2k and Cy7-OLA than Cy7-CA.

The circulation half-lives of both the Cy5.5-NP carrier and Cy7-X model drugs (X=CA, OLA or PLGA2k) for the three different formulations were determined (Supplementary Fig. 7). The loaded Cy7-Xs were cleared from the blood faster than the Cy5.5-NP carriers, indicative of dissociation in the circulation. Model drugs with higher hydrophobicity (Cy7-OLA) or better miscibility (PLGA2k) circulated significantly longer and displayed a half-life closer to that of the Cy5.5-NP carrier, resulting in improved tumour accumulations. The differences in Cy7-Xs' biodistributions suggest they may follow different clearance pathways. The *in vitro* drug exchange experiments presented in Supplementary Fig. 4c show that the hydrophobic Cy7-OLA has high affinity for blood constituents. Therefore, when an OLA-derivatized drug dissociates from the nanoparticle carrier, its subsequent association with albumin and lipoproteins may influence biodistribution, and enhance its circulation half-life and tumour accumulation.

### Drug–nanoparticle compatibility influences antitumour efficacy

**Figure 6:**
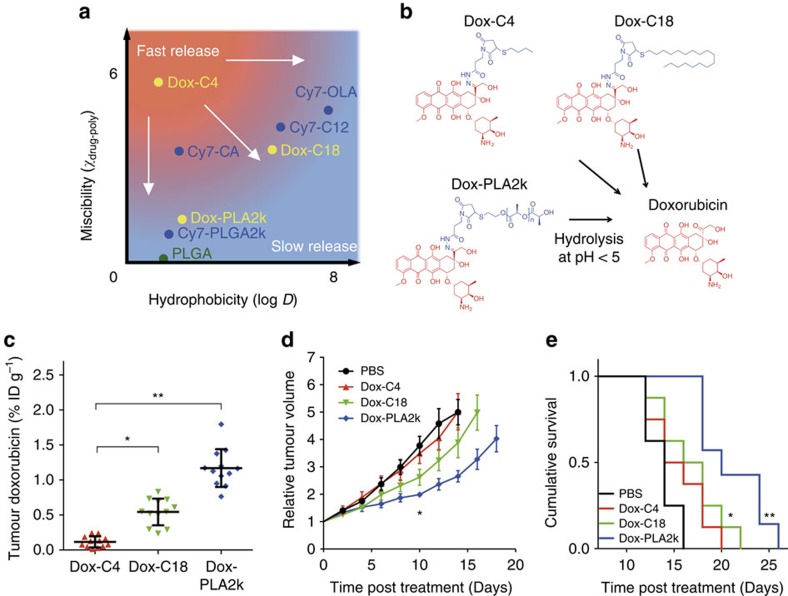
A guideline for efficient drug delivery and its application to doxorubicin nanoparticle therapy. (**a**) Schematic showing that hydrophobicity (log *D*) and miscibility with the PLGA matrix (*χ*_drug-poly_) are two independent parameters that determine the drug's release rate in the circulation. Drugs with properties located in the red area release quickly and those in the blue area release slowly. The scales are arbitrary. White arrows indicate the direction for modifying a parent drug for more efficient drug delivery. (**b**) The pro-drug approach for doxorubicin. In an acidic environment (pH <5), Dox-C4, Dox-C18 and Dox-PLA2k hydrazones will hydrolyse to generate free doxorubicin. (**c**) Doxorubicin concentrations in tumour tissues at 24 h post injection. Three groups of mice (*n*=3–4) were administered NP:Dox-X (X=C4, C18 and PLA2k) at 20 mg doxorubicin equivalent per kg BW. Data are means±s.d. *P* values were calculated with the nonparametric Kruskal–Wallis test and Dunn's nonparametric comparison for *post hoc* testing. *P<0.01, **P<0.001. (**d**,**e**) Therapeutic study with Dox-X nanoparticles. Mice (*n*=8–9) bearing 4T1 tumours were injected with NP:Dox-X at 10 mg doxorubicin equivalent per kg BW or PBS control on days 0, 3 and 6. (**d**) Relative tumour volume up to 18 days post treatment. Data are means±s.e.m. *For values at day 10, *P* values were calculated with the nonparametric Kruskal–Wallis test (*P*=0.001) and Dunn's nonparametric comparison for *post hoc* testing, *P*=0.002 for Dox-PLA2k versus PBS, *P*=0.011 for Dox-PLA2k versus Dox-C4. (**e**) Cumulative mouse survival. *P=0.03 for Dox-C18 versus PBS, **P=0.0001 for Dox-PLA2k versus PBS, using a Log-rank (Mantel–Cox) test.

So far, all the *in vitro* and *in vivo* results suggest that drugs with stronger carrier associations release more slowly in circulation, thereby leading to higher tumour delivery efficiency. On the basis of the findings detailed above, we propose here a guideline for improving drug delivery: as illustrated in Fig. 6a, either increasing the parent drug's hydrophobicity or improving its miscibility (that is, moving from the red zone towards the blue zone) will result in stronger associations, slower drug release in circulation and higher accumulation at the target site.

As a proof of principle, we applied the guideline to the clinical antitumour agent doxorubicin (Dox) in an immune-competent tumour model. We developed three doxorubicin derivatives with distinct physicochemical properties. On the basis of our guideline, Dox-C4 has low hydrophobicity and miscibility, while Dox-C18 has high hydrophobicity and Dox-PLA2k has high miscibility with PLGA (see Fig. 6a and Supplementary Table 1 for basic chemical–physical properties of Dox-X (X=C4, C18 and PLA2k)). Although the different Dox-Xs have different chemical structures (Fig. 6b), they all have the pH-dependent hydrazone moiety that will be cleaved on entering an acidic environment (pH <5), for example, in the lysosomes of tumour cells, consequently generating free doxorubicin^11^,^41^.

This nanoparticle pro-drug concept and the functionality of the resulting doxorubicin were first confirmed *in vitro*. We loaded Dox-X into PLGA–PEG polymeric nanoparticles to generate NP:Dox-X (see characterization in Supplementary Table 2). Fluorescent microscopy studies on 4T1 cells with NP:Dox-X incubations showed their internalization (Supplementary Fig. 8). At early time points, the drug and nanoparticle carriers were entrapped in the cytoplasm. Subsequent doxorubicin release and trafficking to the nucleus occurred at later time points (30 min–24 h)^42^. Importantly, cell viability measurements revealed that NP:Dox-X nanoparticles at the same doxorubicin concentration displayed very similar antitumour activities (Supplementary Fig. 9), indicating that this pro-drug approach does not hamper antitumour efficacy.

To evaluate drug delivery efficiency to the tumour, three groups of BALB/c mice (*n*=3) bearing orthotropic 4T1 breast tumours intravenously received the same (doxorubicin equivalent) dose of NP:Dox-X. Twenty-four hours after injection, we found the doxorubicin concentration in tumour tissues was significantly higher for Dox-PLA2k and Dox-C18 than Dox-C4 (Fig. 6c). To determine the different NP:Dox-Xs' therapeutic efficacy, four groups of mice (*n*=8-9 per group) bearing 4T1 tumours received NP:Dox-Xs at a dose of 10 mg doxorubicin equivalent per kg body weight (BW), or PBS, on days 0, 3 and 6. The relative tumour volumes are plotted in Fig. 6d. Ten days after start of treatment, NP:Dox-C18-treated (*P*=0.028 versus PBS) and NP:Dox-PLA2k-treated mice (*P*<0.0001 versus PBS, *P*=0.006 versus Dox-C4) had significantly smaller tumour volumes compared with NP:Dox-C4- or PBS-treated controls. Correspondingly, the variations in therapeutic efficacy produced different survival curves (Fig. 6e). Treatment with NP:Dox-PLA2k and NP:Dox-C18 resulted in a median survival time of 20 days (*P*<0.0004 versus PBS) and 17 days (*P*<0.05 versus PBS), respectively, compared with 14 days for untreated controls and 15 days for NP:Dox-C4. These results clearly demonstrate that nanoparticles loaded with either Dox-PLA2k, which had improved miscibility, or Dox-C18, which had higher hydrophobicity, improved tumour growth inhibition as compared with Dox-C4.

## Discussion

In the nanomedicine field, drug–carrier compatibility can be improved by modifying the carrier material^25^,^43^, covalently conjugating drugs to the polymers that comprise the nanoparticle core^44^,^45^,^46^,^47^, or by drug derivatization. We here systematically study the latter approach and show that a pro-drug strategy ensures efficient and homogenous encapsulation, which enhances *in vivo* stability and improves nanotherapeutic efficacy. Instead of costly large-scale screening for compatible carrier materials, a rational guideline for pro-drug modification can be employed. This approach represents a potentially valuable strategy in which an optimized nanoparticle carrier can be generically employed for numerous drug classes.

In summary, self-assemblies of poorly water-soluble drugs and polymeric nanoparticles are dynamic structures, which—when exposed to serum—are susceptible to undesired drug release. This is due to drug exchange with plasma proteins, including albumin and high-density lipoprotein, and diminishes the nanoparticle's drug delivery efficiency. Through *in vitro* dynamic experiments, computer simulations, and *in vivo* FRET imaging, we found that drug–carrier compatibility, namely hydrophobicity and miscibility, strongly affects *in vivo* stability and release rate in circulation. On the basis of these findings, we proposed a general guideline for more efficient drug delivery. To test the guideline on a drug currently used in the clinic, we augmented doxorubicin's compatibility with the polymer matrix using pro-drug derivatization technology. This resulted in increased delivery efficiency and improved antitumour efficacy. Thus, our findings prove to be important not only for understanding nanomedicines' *in vivo* fate but also for guiding improvements in nanoparticle drug delivery strategies.

## Methods

### Calculating hydrophobicity and miscibility

Molecules' hydrophobicity was represented by the their distribution coefficients (log *D*) at pH=7.4, which were predicted using the ACD/Labs Percepta Predictor (Advanced Chemistry Development, Inc.). The miscibility between the drug and the polymer matrix was described by Flory–Huggins interaction parameters (*χ*_drug-poly_), which take into account the energy of interspersing polymer with drug molecules, with smaller values indicating better miscibility^25^,^48^. This parameter was calculated using the equation:


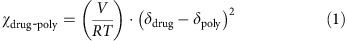


where *V* is the molar volume of the drug molecule, *R* is the gas constant, *T* is the temperature and *δ*_drug_ and *δ*_poly_ are the Hildebrand–Scatchard solubility parameter for drug and the polymer matrix, respectively^49^. The values of *V*, *δ*_drug_ and *δ*_poly_ were calculated through the group contribution method and are described in detail in the Supplementary Methods.

### Synthesizing Cy7-X model drugs loaded nanoparticles

The Cy7-X model drugs were synthesized by conjugating a Cyanine7-NHS ester (Lumiprobe GMBH) and a primary amine, dodecylamine for Cy7-C12, oleylamine for Cy7-OLA and PLGA-NH_2_ 2 kDa for Cy7-PLGA2k. Cy7-CA was used as purchased. The detailed procedures are described in the Supplementary Methods. Cy5.5-conjugated PLGA was synthesized through Steglich esterification between Cy5.5-CA (Lumiprobe) and PLGA (lactide:glycolide, 50:50; molecular weight, 30,000–60,000; Sigma-Aldrich). An estimation of 70% of PLGA chain was conjugated with one Cy7 molecule.

Self-assembled Cy5.5-NP:Cy7-X nanoparticles were synthesized through a nano-precipitaion method. In a typical synthesis, 20 mg PLGA-PEG (molecular weight PLGA 4,000, PEG 2,000; Sigma Aldrich), 4.2 mg Cy5.5-PLGA and a calculated amount of Cy7-X were dissolved in acetonitrile at a concentration of 10 mg ml^−1^. To form nanoparticles, the acetonitrile solution was dripped into 20 ml PBS at a rate of 0.2 ml min^−1^ at room temperature under vigorous stirring. The solution was then continuously stirred for 1 h after dripping to induce evaporation of the organic solvent. The produced nanoparticles were purified through a first centrifugation at 18*g* for 10 min to remove possible aggregates, and then washed at least three times with fresh PBS with centrifugal concentrators (Millipore, 100,000 molecular weight cutoff) and finally concentrated. Nanoparticles were kept at 4 °C and protected from light until use.

### Optical measurement and *in vitro* dynamic experiments

The absorption, emission spectrum and *in vitro* dynamic experiments were performed on a SpectraMax M5e multi-mode microplate reader (Molecular Devices). In a typical dynamic experiment, 2 ml of Cy5.5-NP:Cy7-X solution at 1 mg ml^−1^ was loaded in a quartz cuvette and pre-equilibrated at a controlled temperature in the plate reader for at least 10 min. Then 0.4 ml of FBS (Sigma-Aldrich) or human serum (Type AB, Fisher BioReagents) at 5, 10, 25, 50 or 100% dilution pre-equilibrated at the same temperature was quickly added into the cuvette and well mixed through a pipette within 5 s. The time-dependent fluorescence was recorded in kinetic mode in Cy5.5 channel (*λ*_Exc_=620 nm and *λ*_Emi_=700 nm) and FRET channel (*λ*_Exc_=620 nm and *λ*_Emi_=780 nm) at an interval of 10 s.

### Fast protein liquid chromatography

The FPLC samples were prepared by adding 0.2 ml of 10 mg ml^−1^ solution of Cy5.5-NP:Cy7-X nanoparticles or Cy5.5-Cy7-NP control particles into 0.8 ml of FBS and incubated at 37 °C for 2 h. After incubation, samples were filtrated with a 0.22-μm pore size membrane, and 25 μl of each sample was injected to the FPLC column. The FPLC system was constructed by a Prominence HPLC system (Shimadzu) equipped with a Superose 6, 10/300 GL FPLC column (GE Healthcare Life Sciences). The samples were then eluted with PBS buffer at a flow rate of 0.6 ml min^−1^ while the absorbance of eluted solution was monitored at 190–800 nm by a photodiode array detector. Results were processed and analysed with LC solution software (Shimadzu).

### Computer simulations

To carry out the SMD simulations, first an all-atom simplified version of the block-copolymer system was constructed. This system consisted of 122 polymer chains, 50% of which were PLGA–PEG block-copolymers, whereas the rest were composed of pure PLGA chains. Next, the polymer chains were hydrated with water molecules^50^, and a 40-ns trajectory of unbiased all-atom molecular dynamics simulations (uMD) was performed to equilibrate the entire system. The final stage of the uMD trajectory was used as the starting point for the SMD simulations. The entire system was positioned so that the direction of the reaction pathway (that is, the direction of the steered motion of the drug) was aligned along the *Z*-coordinate. To simplify the simulations, a generic drug-like aromatic compound, that is, phenanthrene, was used to replace Cy7. Different tail parts, CA, C12, OLA and PLGA2k were attached to phenanthrene to modify the overall drug properties along the lines of the experimental protocol. In the SMD simulation, each of these drug models was aligned along the *Z*-coordinate, and force was applied to move the drug models along the reaction coordinate that took them through the different phases of the hydrated block-copolymer systems. The SMD simulations employed the constant velocity algorithm implemented in the NAMD simulation package^51^ and CHARMM force field parameters^52^. The velocity was set to 0.0001 Å ps^−1^ for all the drug models, and the force constant was set to 7.0 kcal mol^−1^ Å^−1^ along the direction of the *Z*-coordinate. A Langevin thermostat and piston were used to maintain the temperature at 300 K and the pressure at 1 atm^53^.

### Cell culture and animal model

Information regarding cell lines (human mammary gland/breast cancer cell line MDA-MB-231, murine mammary gland/breast cancer cell line 4T1) and preparation of animal models can be found in the Supplementary Methods. All animal-handling protocols were approved by the Icahn School of Medicine at Mount Sinai Institutional Animal Care and Use Committee.

### Intravital microscopy

Preparation of the window chamber mouse model is described in the Supplementary Methods. The imaging experiment was performed on a Leica SP8 confocal laser scanning microscopy using an HC/PL/APO × 10 air objective with 0.4 numerical aperture. Directly after injection of Cy3.5-NP or Cy3.5-NP:Cy5-X (X=C12, OLA and PLGA2k), focus was adjusted to the tumour vasculature that was subsequently imaged. For spectral imaging, 4 mice were subjected to 45 min of sequential spectral imaging at a temporal resolution of 12 s and spatial resolution of 2.27 μm^2^ with the filter sets *λ*_Exc_=570 nm and *λ*_Em_=591, 602, 613, 624, 635, 646, 657, 668, 679, 690 and 701 nm. For high-spatial resolution imaging (1.13 μm^2^), another 4 mice were subjected to 60-min sequential imaging at a temporal resolution of 3 min. Three optical channels were recorded: Cy3.5 (*λ*_Exc_=570 nm and *λ*_Emi_=585–620 nm); FRET (*λ*_Exc_=570 nm and *λ*_Emi_=655–730 nm); and Cy5 (*λ*_Exc_=633 nm and *λ*_Emi_=655–730 nm). The images recorded were analysed using Fiji/ImageJ2 software. The window chamber experiments were approved by the institutional ethics committee and were in accordance with national (Norway) and institutional (NTNU) guidelines.

### *In vivo* near-infrared fluorescence imaging

Nude mice bearing MDA-MB-231 tumour xenografts on their right flanks were used, which enables *in vivo* optical imaging of these superficial tissues. Four groups of tumour-bearing female nude mice (*n*=8) were administrated through tail veins with Cy5.5-NP:Cy7-X (X=CA, OLA and PLGA2k) or Cy5.5-NP control at a dose of 1.5 g polymer per kg BW. NIRF imaging was performed using a Xenogen IVIS Spectrum imaging system (Perkin Elmer) for each group of mice at selected post-injection time points (*t*=0.5, 1, 1.5, 2, 3, 4, 5, 6, 7, 8, 10, 12, 19, 24, 28, 32, 36 and 48 h). Three optical channels were recorded with selected excitation and emission band-pass filters: Cy5.5 (*λ*_Exc_=640±18 nm and *λ*_Em_=720±10 nm); Cy7 (*λ*_Exc_=745±18 nm and *λ*_Em_=800±10 nm); and FRET (*λ*_Exc_=640±18 nm and *λ*_Em_=800±10 nm). The exposure time for each image was 2 s. During the imaging, mice were anaesthetized with vapourized isoflurane administered at 1.5% via a nose cone. Results were processed and analysed using Living Image software (PerkinElmer) by drawing a region of interest in the tumour area.

### Synthesis of therapeutic nanoparticles

Doxorubicin derivatives were synthesized through the conjugation of aldoxorubicin (Medkoo Bioscience) with a thiol:butanethiol (Sigma-Aldrich) for Dox-C4, 1-octadecanethiol (Sigma-Aldrich) for Dox-C18 and poly(L-lactide) thiol (Mn 2,500; Sigma-Aldrich) for Dox-PLA2k. Dox-X-loaded nanoparticle NP:Dox-X were synthesized through a nanoprecipitation method similar to the synthesis of Cy5.5-NP:Cy7-X. The detailed procedures are described in the Supplementary Methods.

### Doxorubicin tissue distribution in tumour mouse model

Four groups of BALB/c mice bearing orthotropic 4T1 tumour (*n*=3) were injected with either NP:Dox-X (X=C4, C18, PLA2k) at 20 mg Dox equivalent per kg BW or the PBS control through the tail vein. At 24 h post injection, the animals were killed, and 40 ml of PBS was perfused through the heart left ventricle. Tumours were collected and kept on ice throughout the extraction procedure. This procedure was slightly modified from existing literature^11^,^54^. Samples (∼100 mg) from tumours were collected, weighed and cut into small pieces, and loaded into Lysing Matrix D microtubes (MP Biomedicals), each of which was preloaded with 400 mg RIPA cell lysis buffer (Sigma-Aldrich) and collagenase enzyme. After 40 min incubation at 37 °C, the digested tissue samples were homogenized using 1.4 mm ceramic spheres and a Fastprep-24 benchtop homogenizer (MP Biomedicals) for 60 s at 6.5 m s^−1^. For each sample, 200 μl homogenized solution was transferred to a microtube, then 50 μl Triton X-100 solution (10% v/v; Sigma-Aldrich) and 750 μl acidified isopropanol (0.75 M HCl) were added in sequence to extract doxorubicin. After incubating overnight at 4 °C in darkness, the extraction solution was vortexed and then centrifuged at 14,000 r.p.m. for 10 min. Then 200 μl of the supernatant and standard doxorubicin solutions (0.1–50 μg ml^−1^, prepared in acidified isopropanol) were placed in a 96-well plate, and doxorubicin fluorescence was measured using a microplate reader (*λ*_Exc_=485 nm and *λ*_Exc_=590 nm). To correct the background auto-fluorescence, the tissues of tumour or organs from the control mice were processed similarly, and the auto-fluorescence from the blank tissue samples was recorded. That amount was subtracted from the doxorubicin-containing samples. Mann–Whitney test was performed on the doxorubicin concentration in tissue from different groups, and two-tailed *P* values were obtained using GraphPad Prism version 6.0 (GraphPad Software).

### Therapeutic treatment and inhibition of tumour growth

Female BALB/c mice (6–8 weeks old) bearing orthotropic 4T1 tumours were checked every 2 days to monitor tumour progression and BW. The tumour volume was calculated using the following formula:


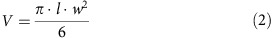


where *l* and *w* are the length (maximum diameter) and width (minimum diameter) of the tumour, respectively, measured using digital calipers. When tumour volume reached around 200 mm^3^, mice were randomized into four groups (*n*=8–9) by tumour size and BW, and treated via tail vein with PBS control or NP:Dox-X (X=C4, C18 and PLA2k) at 10 mg doxorubicin equivalent per kg BW on days 0, 3 and 6. Tumour dimensions and BW were monitored every 2 days. For tumour volumes at day 10, significance of differences was calculated by use of the nonparametric Kruskal–Wallis test and Dunn's nonparametric comparison for *post hoc* testing of between group differences. Mice with tumour volumes >1,000 mm^3^ were killed. The cumulative survival curves were compared using Kaplan–Meier analysis and Log-rank (Mantel–Cox) test. Probability values of *P*<0.05 were considered significant. Statistical analyses were performed using SPSS (Statistical Package for the Social Sciences) version 22.0 and GraphPad Prism version 6.0.

## Additional information

**How to cite this article:** Zhao, Y. *et al*. Augmenting drug–carrier compatibility improves tumour nanotherapy efficacy. *Nat. Commun.* 7:11221 doi: 10.1038/ncomms11221 (2016).

## Supplementary Material

Supplementary Information Supplementary Figures 1-9, Supplementary Tables 1-3, Supplementary Discussion, Supplementary Methods and Supplementary References
