# Supplementary material for: Augmenting drug–carrier compatibility improves tumour nanotherapy efficacy
Source: Nat Commun. 2016 Apr 13;7:11221. doi: 10.1038/ncomms11221 (PMC4833858; doi:10.1038/ncomms11221)
Supplement: Supplementary Information — Supplementary Figures 1-9, Supplementary Tables 1-3, Supplementary Discussion, Supplementary Methods and Supplementary References [file ncomms11221-s1.pdf]

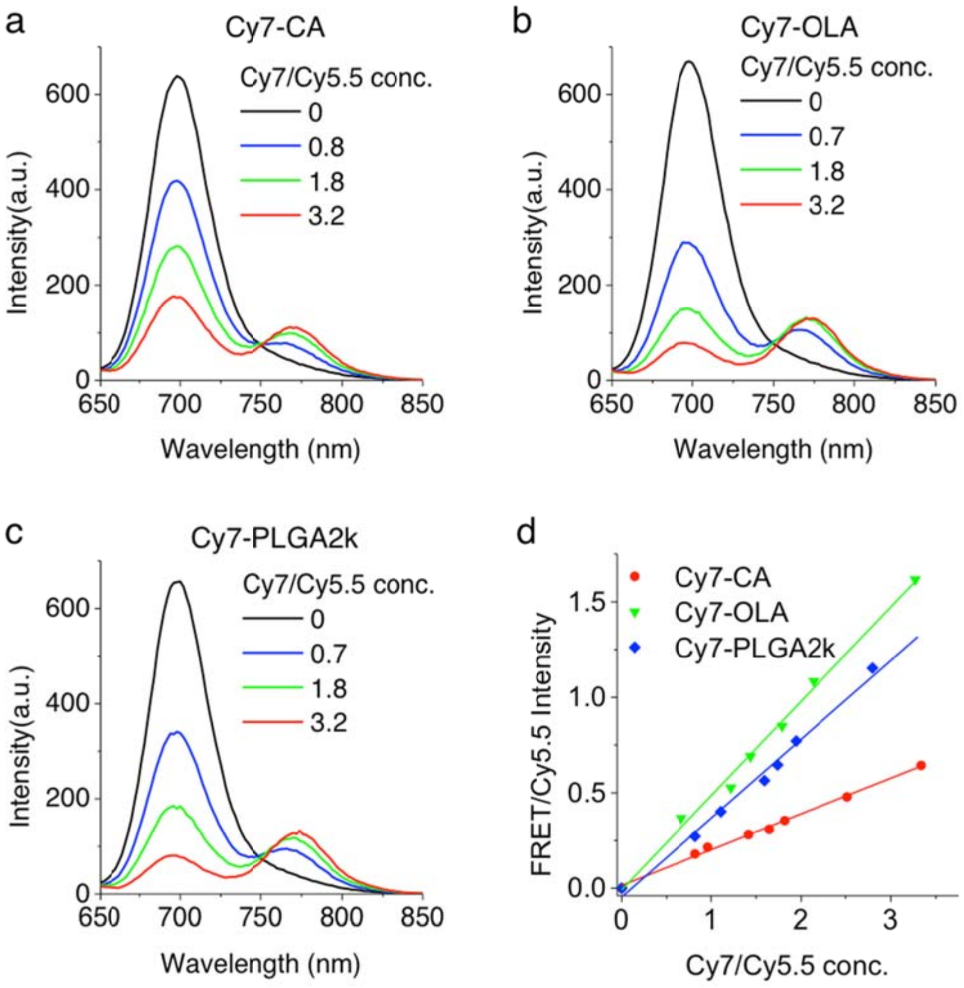

Supplementary Figure 1. The correlation between emission spectra and the loading content of drug models. a-c, The emission spectra of Cy5.5-NP carrier particles (labeled with fixed amount of Cy5.5 per particle) loaded with an increasing amount of Cy7-CA (**a**), Cy7-OLA (**b**), or Cy7-PLGA2k (**c**). The contents of the Cy7 in different particles are presented as Cy7/Cy5.5 concentration ratio determined by Cy5.5 and Cy7 absorbance. **d**, The emission peak ratio of FRET/Cy5.5 (768 nm/698 nm) for each particle is plotted against the concentration ratio of Cy7/Cy5.5. Solid lines are the liner fits. The higher slope of the linear fit for Cy7-OLA and Cy7-PLGA2k may be due to a shorter average donor-acceptor distance, thereby indicating that Cy7-OLA and Cy7-PLGA2k are located closer to the center of the particle than Cy7-CA.

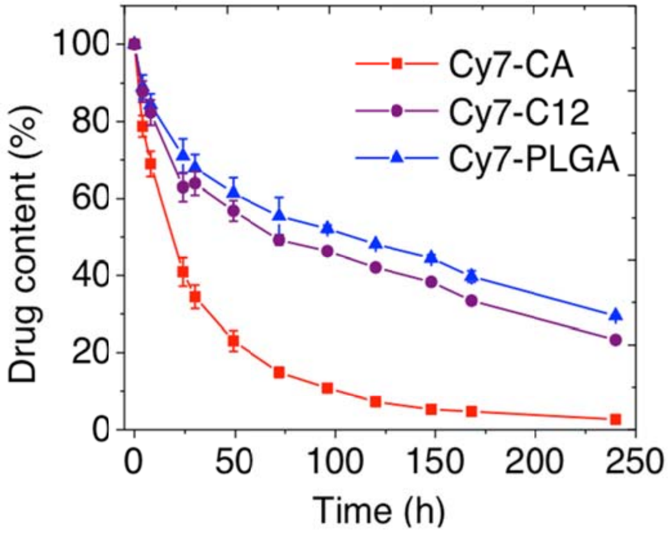

Supplementary Figure 2. The *in vitro* release of drug models in PBS incubated at 37 °C. Drug model remainders in the samples were determined by Cy7 absorbance (n=3). The half-life for Cy7-CA, Cy7-C12, and Cy7-conjugated on PLGA are about 22, 75, and 105 hours, respectively.

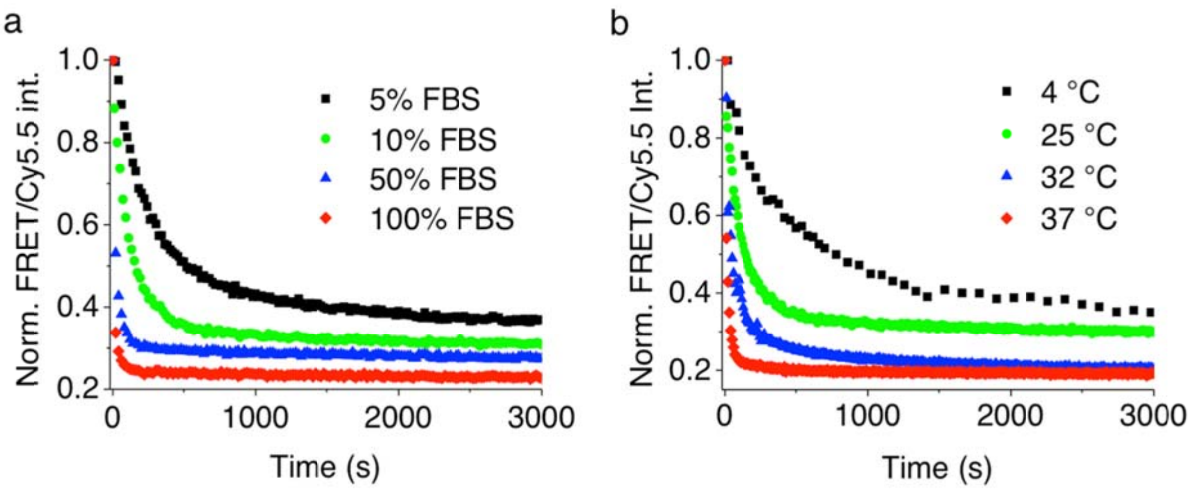

Supplementary Figure 3. Concentration and temperature dependence of drug release in the presence of FBS. For dynamic experiments, a fixed amount of Cy5.5-NP:Cy7-CA was mixed with either **a**, an indicated dilution of FBS at 25 °C or **b**, 10% FBS at indicated temperatures.

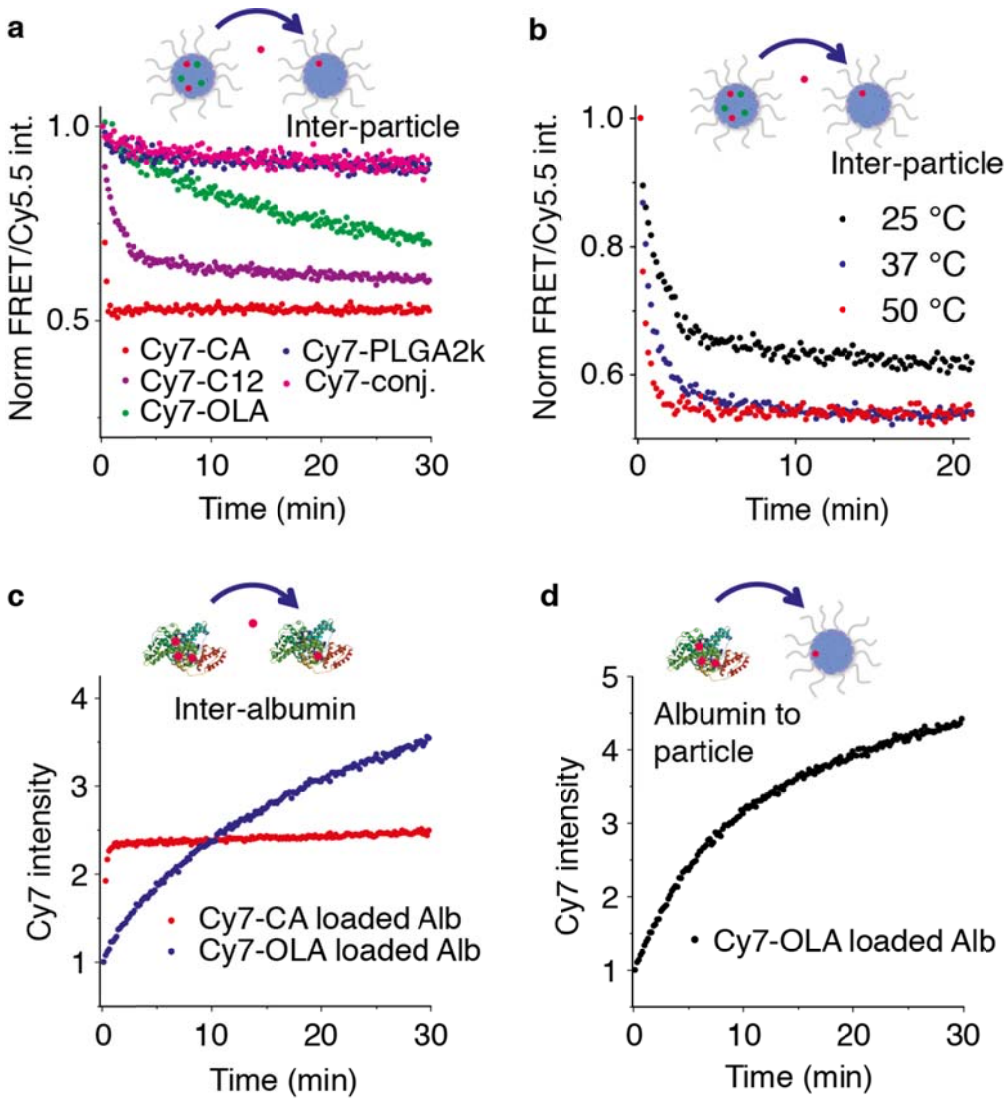

Supplementary Figure 4. Additional drug exchange dynamic experiments. a, Cy5.5-NP:Cy7-X (X=CA, C12, OLA, PLGA2k) or control Cy5.5-Cy7-NP were mixed with blank PLGA-PEG nanoparticlesat 25 °C. **b**, Cy5.5-NP:Cy7-C12 was mixed with blank PLGA-PEG micelles at indicated temperatures. **c,** Albumin pre-loaded with Cy7-CA or Cy7-OLA drug models (Cy7 intensity is self-quenched at this state) were mixed with blank albumin solution in PBS at 25 °C. Cy7 intensity increased during dequenching, indicating drug exchange between albumin. **d**, Albumin pre-loaded with Cy-OLA was mixed with blank PLGA-PEG micelles at 25 °C.

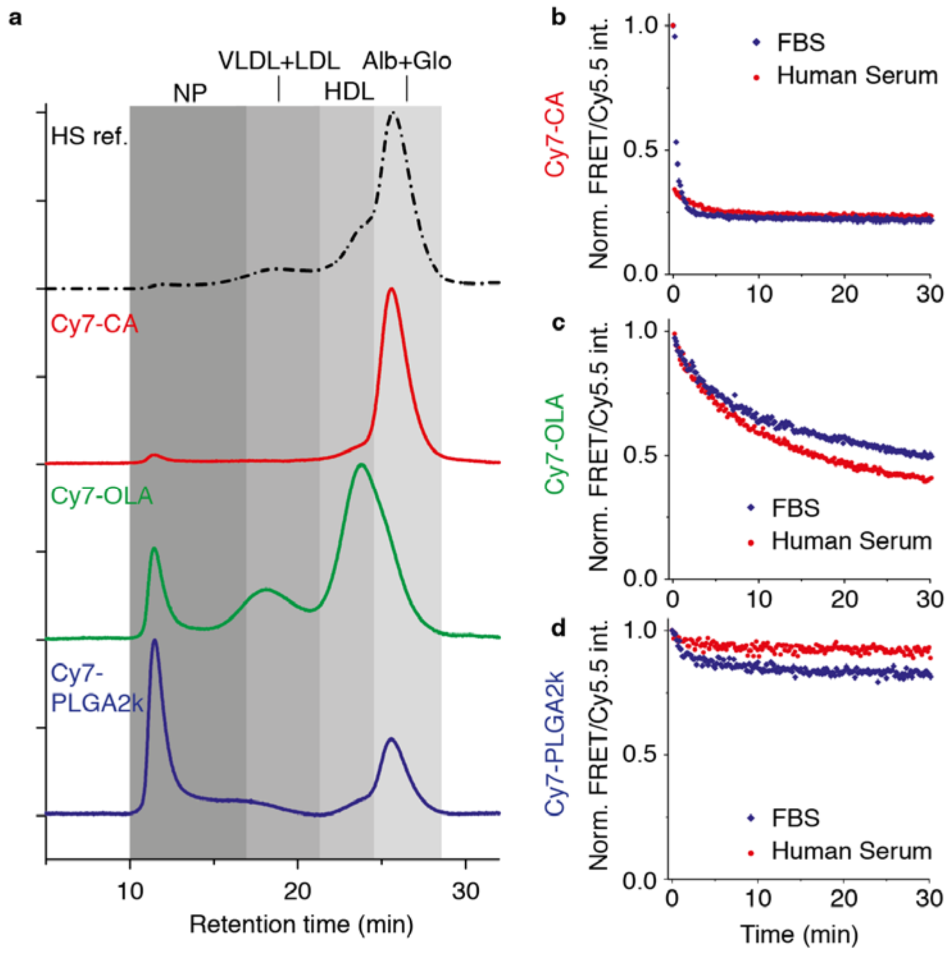

Supplementary Figure 5. ***In vitro*** **drug relase experiment of Cy5.5-NP:Cy7-X (X=CA, OLA, PLGA2k) in human serum(HS) compared to fetal bovine serum (FBS). a,** FPLC analysis of Cy7-X distribution in incubation mixtures of FBS and Cy5.5-NP:Cy7-X. Chromatograms of the HS reference and Cy7-X were recorded through absorbance at 250 nm and 760 nm, respectively. In general, Cy7-X has very similar distribution in HS compared to in FBS (Fig. 2e). Cy7-OLA has higher binding in the HDL and LDL fraction possible due to higher content of lipoproteins in HS. **b-d**, Comparison of drug release in FBS and in HS. The normalized FRET/Cy5.5 intensity ratio is ploted against time after mixing. The releasing rate in HS also follows the order CA>OLA>PLGA2k. The Cy7-OLA releasing in HS is slightly faster than that in FBS possibly also due to higher lipropotein content in HS.

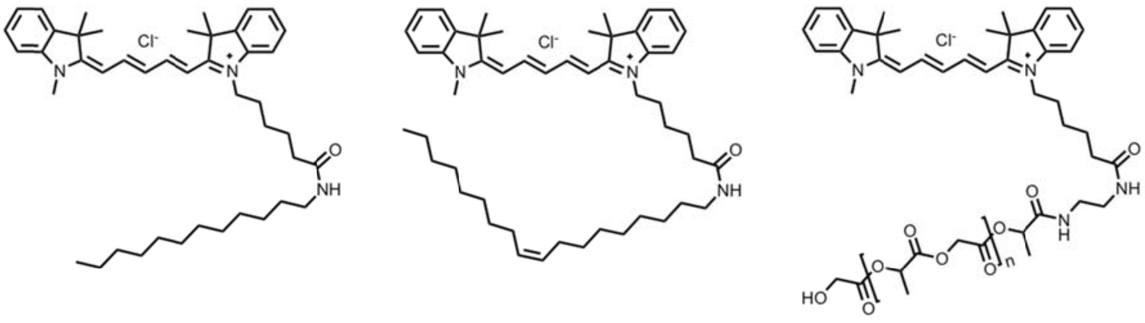

Cy5-C12 Cy5-OLA Cy5-PLGA2k

Supplementary Figure 6. Chemical structures of the Cy5-X (X=C12, OLA, PLGA2k) model drugs.

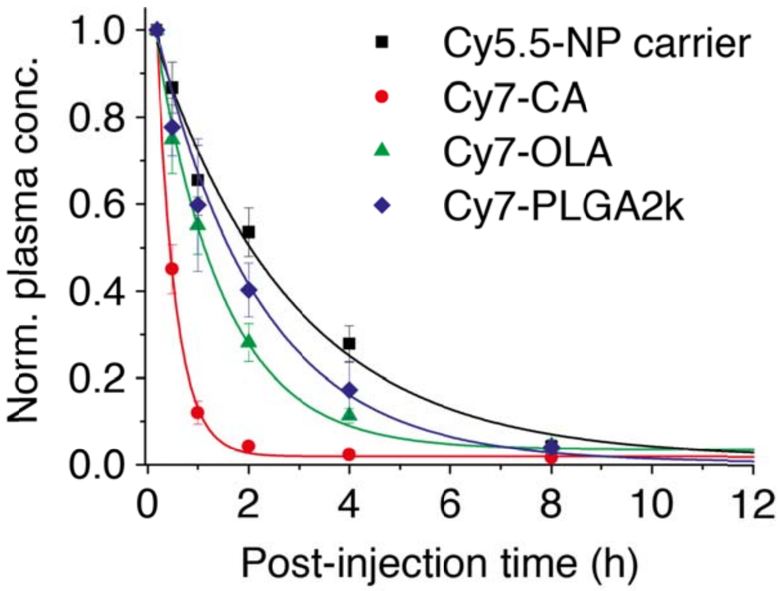

Supplementary Figure 7. Plasma pharmacokinetics of individual components of Cy5.5-NP:Cy7-X (X=CA, OLA, PLGA2k). Cy7-X or Cy5.5-NP carrier concentrations in plasma, determined by Cy7 and Cy5.5 absorbance, are plotted against post-injection time. A two-phase decay model was used to fit each group of data, which yielded an average half-life of 0.37 h for Cy7-CA, 1.34 h for Cy7-OLA, 2.36 h for Cy7-PLGA2k, and 2.72 h for the carrier nanoparticles.

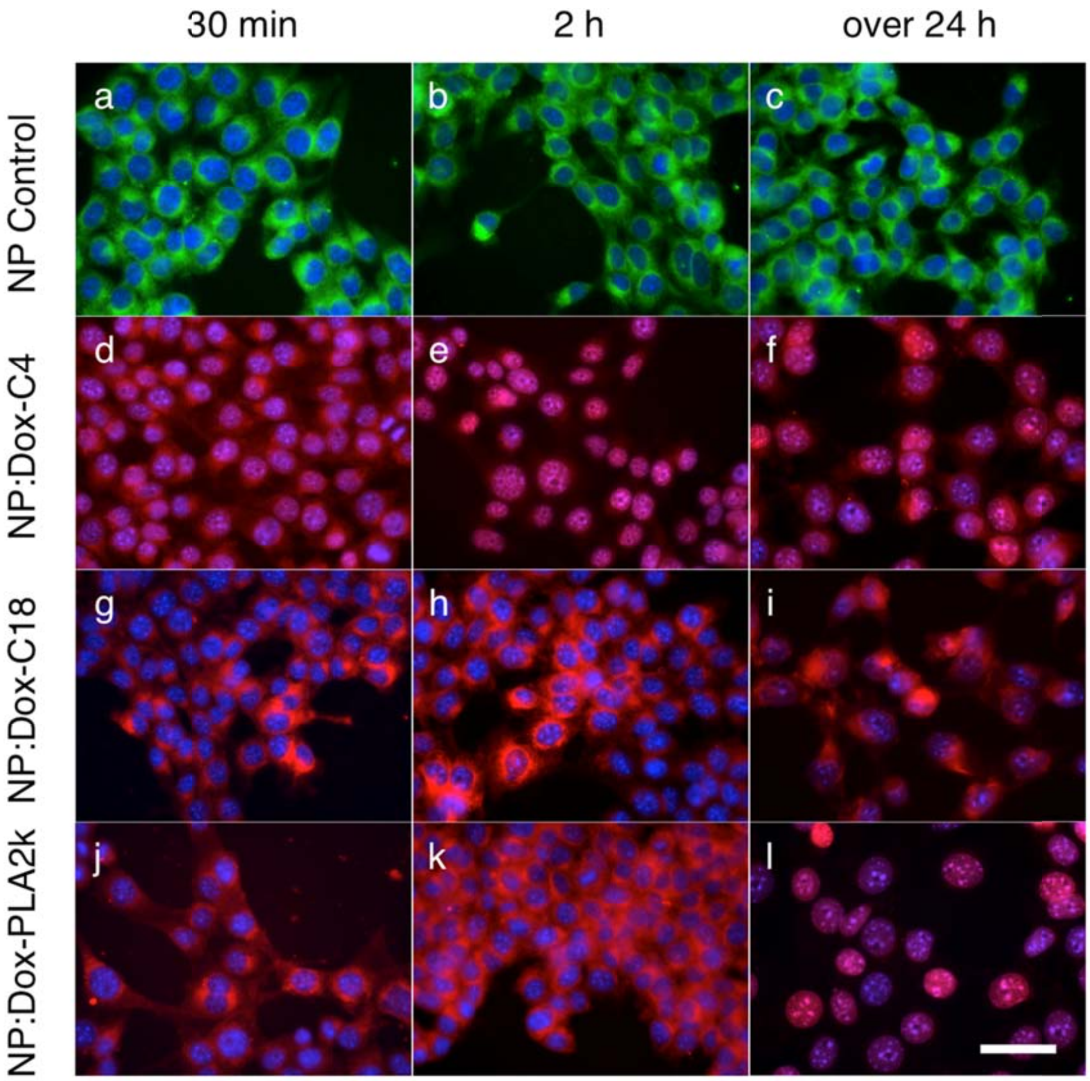

Supplementary Figure 8. Fluorescence microscopy images of cell internalization and subsequent drug delivery to the nucleus. 4T1 cells were incubated with Cy3.5-NP carrier particles control (**a**-**c**), NP:Dox-C4 (**d**-**f**), NP:Dox-C18 (**g**-**i**), and NP:Dox-PLA2k (**j**-**l**). Cy3.5-NP (green) were internalized and stayed in cytoplasm at all times. Dox-Xs (red) were internalized with the nanoparticle in the cytoplasm at earlier time points. At later time points, doxorubicin was generated and diffused into the cell nucleus (blue). Scale bar for all images is 50 μm.

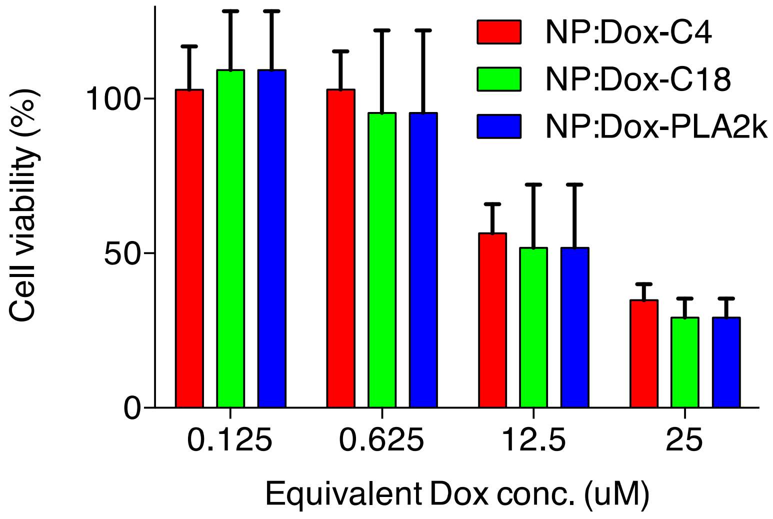

Supplementary Figure 9. Cell viability for NP:Dox-X (X=C4, C18, PLA2k) in 4T1 cells (n=6). No significant differences in cell viability were found among NP:Dox-X at all concentrations.

Supplementary Table 1. Basic physicochemical properties of drug models and drug molecules used in the current study.

Molecules	[1]Hydrophobicity log D (pH=7.4)	[2]Intrinsic solubility Log S_0	[3]Molar volume V (cm^3/mol)	[4]Solubility parameter δ	[5]Flory-Huggins Parameter $\chi_{d\text{-}p}$
**Cy7-CA**	2.48	-8.17	470	19.9	1.28
**Cy7-C12**	7.26	-11.86	661	19.0	3.27
**Cy7-OLA**	7.74	-13.03	769	18.6	4.72
**Cy7-PLGA2k**	1.75	-7.64	1761	22.0	0.18
**Cy5-CA**	1.96	-6.62	419	19.6	1.42
**Cy5-C12**	6.53	-11.16	610	18.7	3.55
**Cy5-OLA**	6.73	-11.65	718	18.3	5.11
**Cy5-PLGA2k**	1.44	-7.66	1710	22.0	0.17
**Doxorubicin**	-1.04	-3.2	278	31.1	8.3
**Dox-C4**	0.57	-6.38	469	28.4	6.58
**Dox-C18**	6.95	-12.75	694	24.9	1.61
**Dox-PLA2k**	2.89	-10.28	2186	22.7	0.04
**PLGA**	1.69	-2.78	-	22.5	0
**PLA**	0.49	-4.56	-	21.5	0

1. The molecule's hydrophobicity is evaluated by the distribution coefficient (log D) at physiological pH =7.4. 2. Intrinsic solubility S_0 is the equilibrium solubility of the free acid or base form of an ionizable compound at a pH where it is fully unionized. log D and log S_0 values are predicted using a computer program. 3. Molar volume is the volume occupied by one mole of substance. 4. Hildebrand-Scatchard solubility parameter is calculated through group contributions using Hoftyzer and Van Krevelen methods. 5. Calculated by comparing solubility parameters of drug and polymer matrix.

Supplementary Table 2. Characterizations of PLGA-PEG nanoparticles used in the current study.

Nanoparticles	[1]Mean Size D_n (nm)	[2]Mean Size D_v (nm)	PDI	Zeta potential (mV)
**Cy5.5-NP:Cy7-CA**	48.9	74	0.17	-8.4
**Cy5.5-NP:Cy7-C12**	42.5	65	0.17	-18
**Cy5.5-NP:Cy7-OLA**	47.9	71.3	0.16	-9.3
**Cy5.5-NP:Cy7-PLGA2k**	46.2	66.3	0.15	-10
**NP:Dox-C4**	21.3	27.3	0.17	-6.8
**NP:Dox-C18**	18.8	31.6	0.21	-13.8
**NP:Dox-PLA2k**	26.9	54.2	0.20	-8.7

1. Number-weighted. 2. Volume-weighted mean diameters determined through dynamic light scattering.

Supplementary Table 3. [1] Fitting results for the decay curves of dynamic experiments.

Nanoparticles (Cy5.5-NP+)	FBS Dilutions	Temperature (°C)	T_1 (s)	A_1	T_2 (s)	A_2	T_{avg} (s)
**Cy7-CA**	100%	37	17	0.91	403	0.09	51
**Cy7-C12**	100%	37	48	0.89	804	0.11	131
**Cy7-OLA**	100%	37	402	0.39	2634	0.61	1763
**Cy7-CA**	100%	25	36	0.88	1946	0.12	256
**Cy7-C12**	100%	25	66	0.78	937	0.22	257
**Cy7-OLA**	100%	25	686	0.31	3646	0.69	2728
**Cy7-CA**	10%	37	23	0.94	408	0.06	46
**Cy7-C12**	10%	37	30	0.71	506	0.29	168
**Cy7-OLA**	10%	37	-	-	937	-	937
**Cy7-CA**	10%	25	112	0.88	981	0.12	216
**Cy7-C12**	10%	25	169	0.83	1186	0.17	341
**Cy7-OLA**	10%	25	-	-	2130	-	2130

1. FRET/Cy5.5 intensity ratio decay curves were fitted with bi-exponential decay functions. T_1 and T_2 are half-life of the fast and slow component; A_1 and A_2 are their amplitude. T_{avg} are the average half-lives calculated through: $T_{avg}=A1*T_1 + A2*T_2$.

Supplementary Discussion

Additional dynamic experiments

Additional dynamic experiments were performed to further investigate the phenomenon of drug molecules exchanging between nanocarriers. To check if the drug molecules are able to exchange between PLGA-PEG nanocarriers, Cy5.5-NP:Cy7-X (X=CA, C12, OLA, and PLGA2k) and control particles (both Cy5.5, Cy7 conjugated to PLGA core) were mixed with blank PLGA-PEG nanoparticles and the FRET dynamics were recorded. We observed increased Cy5.5 intensity and correspondingly decreased FRET intensity similar to the results of the FBS mixing dynamic experiment. As presented in Supplementary Fig. 4a, the decreased FRET/Cy5.5 intensity ratio (FRET ratio) indicates Cy7-X release. Besides similar concentration and temperature dependence (Supplementary Fig. 4b), the exchange rate also varies for drugs with different properties, following a similar order to the drug release in FBS: CA>C12>OLA>PLGA2k. These similarities suggest drug exchanging between particles likely uses the same mechanism as the drug exchange between particles and FBS.

The next question is whether drug molecule association with nature carrier protein is a reversible process. To that end, we preloaded the albumin with Cy7-X (X=CA, OLA) molecules by dripping an acetonitrile solution of free Cy7-X into albumin solution in PBS under vigorous stirring, followed by purification through centrifugal filtration. When albumin was loaded with multiple Cy7-X molecules, the high concentration of localized dye molecules caused Cy7 fluorescence self-quenching.

After mixing the Cy7-X-loaded albumin with blank albumin, we observed gradually increased Cy7 intensity due to dequenching when Cy7-X molecules were far apart (Supplementary Fig. 4c), thereby indicating Cy7-X exchange and relocation from the originally-attached albumin to the newly-added blank albumin. A faster increase in Cy7-OLA intensity compared to Cy7-CA demonstrates that Cy7-OLA bonds more strongly to the albumin. This experiment shows that the Cy7-X molecule's association with the albumin is reversible, and the activation energy is higher for more hydrophobic molecules. In the last dynamic experiment, Cy7-CA-loaded albumin was mixed with blank PLGA-PEG micelles. Increased Cy7 fluorescence indicated that the Cy7-CA attached to albumin could be exchanged onto the PLGA-PEG micelles (Supplementary Fig. 4d).

Based on our various dynamic experiments, we reached several conclusions: (1) The exchange of drug molecules between the synthesized carriers and nature carriers is a fully reversible process. (2) Drug molecules will migrate from drug-rich carriers to drug-poor carriers until finally reaching a thermodynamic equilibrium. (3) The exchange rate depends on temperature, carrier concentration, and the association energy between the drug molecules and the carrier particles.

Drug molecule exchange mechanism

Computer simulation results suggest that when the drug molecules have low miscibility with the polymer matrix, they localize at the interface of PLGA and PEG/water, instead of incorporating in the PLGA matrix. In this case, the

nanoparticle interfaces act as hydrophobic pockets where poorly water-soluble drugs can absorb to and detach from freely, activated by the thermal fluctuation.

The mechanism of drug exchange can be expressed in a simple equation for a first order reaction (e.g. between nanoparticle and albumin):

$$NP^{*} + Alb \underset{k^{-}}{\overset{k^{+}}{\rightleftharpoons}} NP + Alb^{*} \tag{1}$$

where the star denotes the absorption of drug molecules on NP or albumin, and k^+ ad k^- are the forward and backward reaction constant. Since the dissociation is a temperature-dependent process, the Arrhenius equation can be used to express this relation:

$$k^{+} = Ae^{-\frac{E_{NP}}{k_B T}} \tag{2}$$

$$k^{-} = Ae^{-\frac{E_{Alb}}{k_B T}} \tag{3}$$

where A is a prefactor, k_B is Boltzmann constant, T is temperature in Kelvin, and E_{NP}, E_{Alb} are the activation energy for dissociation of drug molecules from nanoparticle or albumin, respectively. For drug molecules with higher hydrophobicity or better miscibility, E_{NP} is estimated to be higher and thus a slower dissociation rate is expected.

Supplementary Methods

Synthesis of Cy7-X (X= CA, C12, OLA, PLGA2k) fluorescence-labeled model drugs

Cy7-CA (Cyanine7 carboxylic acid) was used as purchased (Lumiprobe GMBH, Hannover, Germany). The Cy7-X model drugs were synthesized by conjugating a Cyanine7-NHS ester (Lumiprobe GMBH) and a primary amine. The amines used for the synthesis were dodecylamine (≥99%, Sigma-Aldrich, St. Louis, MO, USA) for Cy7-C12, oleylamine (≥98%, Sigma-Aldrich) for Cy7-OLA, and Poly (lactic-co-glycolic)-NH_2 2kDa succinic acid dihydrazide (Mn=2470, PDI=1.59 analysis from the manufacture, Akina Inc., West Lafayette, IN, USA) for Cy7-PLGA2k. The reaction was performed by mixing Cy7-NHS ester and the amine (1:1.1 in molar ratio) in anhydrous dichloromethane (≥99.8%, Sigma-Aldrich) and stirring overnight at room temperature in darkness. The reaction was monitored by thin layer chromatography (TLC; eluted by chloroform/methanol, 5:1, v/v). After the reaction, the solvent was removed through rotavapor, and the residue was redissolved in acetonitrile (99.8%, Sigma-Aldrich). The unreacted amine was removed via filtration, and the product in acetonitrile was stored at -20 °C in darkness for further use. The success of conjugation was confirmed through LC/MS mass spectrometry (Agilent, Santa Clara, CA, USA).

Calculating solubility parameter, molecular volume, and Flory-Huggins parameter

The total molar volume V in $cm^3 mol^{-1}$ was calculated through the group contribution method according to Fedors method.[1] Using Hansen's approach, the total solubility

parameter can be divided into three partial solubility constants, each of which describes a different type of intermolecular interaction:

$$\delta_{total}^2 = \delta_d^2 + \delta_p^2 + \delta_h^2 \quad (4)$$

where δ_d, δ_p, and δ_h are the partial solubility parameters for van der Waals dispersion forces, dipole-dipole interaction, and hydrogen bonding, respectively. Those parameters can then be estimated from group contributions using Hoftyzer and Van Krevelen method, using the following equations:

$$\delta_d = \frac{\sum F_{di}}{V} \quad (5)$$

$$\delta_p = \frac{\sqrt{\sum F_{pi}^2}}{V} \quad (6)$$

$$\delta_h = \sqrt{\frac{\sum E_{hi}}{V}} \quad (7)$$

where F_{di} and F_{pi} are the additive molar attraction constants, and E_{hi} is the additive cohesive energy for the groups being considered. The values of F_{di} , F_{pi}, and E_{hi} for each group can be found in literature.[2]

Synthesis and characterization of Cy5.5-NP:Cy7-X (X= CA, C12, OLA, PLGA2k) nanoparticles

Cy5.5-conjugated PLGA was synthesized through Steglich esterification between Cy5.5 carboxylic acid (Lumiprobe, GMBH) and poly(D,L-lactide-co-glycolide) (PLGA; lactide:glycolide, 50:50, Mw 30k-60k; Sigma-Aldrich). An estimation of 70% of PLGA chain was conjugated with one Cy7 molecule. Detailed descriptions can be found

elsewhere.[3] Self-assembled Cy5.5-NP:Cy7-X nanoparticles were synthesized through a nanoprecipitaion method. In a typical synthesis, 20 mg Poly(ethylene glycol) methyl ether-block-poly(lactide-co-glycolide) (PLGA-PEG; PLGA Mn 4000, PEG Mn 2000; Sigma-Aldrich), 4.2 mg Cy5.5-PLGA, and a calculated amount of Cy7-X were dissolved in acetonitrile at a concentration of 10 mg ml^{-1}. To form nanoparticles, the acetonitrile solution was dripped into 20 ml PBS at a rate of 0.2 ml min^{-1} at room temperature under vigorous stirring. The solution was then continuously stirred for 1 h after dripping to induce evaporation of the organic solvent. The produced nanoparticles were purified through a first centrifugation at 18.0g for 10 min to remove possible aggregates and then washed at least 3 times with fresh PBS with centrifugal concentrators (Millipore, 100k molecular weight cut off (MWCO)) and finally concentrated. Nanoparticles were kept at 4 °C and protected from light until use.

The hydrodynamic sizes of the Cy5.5-NP:Cy7-X nanoparticles were determined by dynamic light scattering (DLS) on a ZetaPALS instrument (Brookhaven Instruments Coorporation). These particles' zeta potentials were determined by zeta potential analyzer using the same instrument. Each sample was measured 5 times, and an average value of the results was used.

Optical measurement and in vitro dynamic experiment

The absorption, emission spectrum, and *in vitro* dynamic experiments were performed on a SpectraMax M5e multi-mode microplate reader (Molecular Devices,

Sunnyvale, CA, USA). Samples were measured in 96-well plates or a 3.5 ml standard quartz cuvette. Emission spectra of Cy5.5-NP:Cy7-X was recorded in a resolution of 2 nm with excitation at 620 nm and a long pass filter at 630 nm. In a typical dynamic experiment, 2 ml of Cy5.5-NP:Cy7-X solution at 1 mg ml^{-1} was loaded in a quartz cuvette and pre-equilibrated at a controlled temperature in the plate reader for at least 10 min. Then 0.4 ml of fetal bovine serum (FBS; Sigma-Aldrich) at 5%, 10%, 25%, 50%, or 100% dilution, pre-equilibrated at the same temperature, was quickly added into the cuvette and well mixed through a pipette within 5 seconds. The time-dependent fluorescence was recorded in kinetic mode in three optical channels: Cy5.5 (λ_{Exc} = 620 nm, λ_{Emi} = 700 nm, cut-off at 630 nm), Cy7 (λ_{Exc} = 740 nm, λ_{Emi} = 800 nm), and FRET (λ_{Exc} = 620 nm, λ_{Emi} = 780 nm, cut-off at 695 nm) with medium gain on PMT and 6 flashes per read. The Cy7 channel was recorded to monitor self-quenching effect of Cy7-X on the particles. The samples showed no significant increase of Cy7 intensity after mixing with FBS. Recording started 20 seconds before mixing and continued until 1-2 hours post-mixing with a 10-second interval between each measurement. The control experiment was performed by mixing Cy5.5-NP:Cy7-X solution and PBS in the same fashion. No obvious photobleaching of the fluorescent dyes was observed in the dynamic experiments.

Cell culture and animal model

Human mammary gland/breast cancer cell line MDA-MB-231 (ATCC HTB-26) was obtained from American Type Culture Collection (Manassas, VA USA) and was

cultured at 37 °C under 5% CO_2 in Dulbecco's modified eagle medium (DMEM) supplemented with 10% FBS and Penicillin-Streptomycin. Murine mammary gland/breast cancer cell line 4T1 was a kind gift from Thomas Reiner's laboratory at Memorial Sloan Kettering Cancer Center (New York, NY, USA) and were cultured at 37 °C under 5% CO_2 in DMEM culture medium supplemented with 10% FBS and Penicillin-Streptomycin.

Six-week-old female NCR/NU mice were obtained from Charles River Laboratories. (Wilmington, MA, USA) and were supplied with water and a standard rodent chow diet *ad libitum*. The tumor was established by injecting $3x10^6$ MDA-MB-231 cells in 50 µl of serum-free DMEM on the mouse's right side flank. Imaging and bio-distribution experiments were preformed 30-40 days after tumor implantation. The animal diet was changed to AIN-93 M maintenance purified diet (TestDiet, Richmond, IN, USA) 1 week before the imaging experiment to reduce the autofluorescent background.

4-5 week old female BALB/cAnNCrl mice were obtained from Charles River Laboratories, and were supplied with water and a standard rodent chow *ad libitum*. The tumor was induced by injecting $1x10^6$ 4T1 cells in 50 µl of serum-free DMEM in the right mammary line of the mice. All animal handling was approved by the Icahn School of Medicine at Mount Sinai Institutional Animal Care and Use Committee (IACUC).

Intravital microscopy

The window chamber mouse model was prepared according to previously described procedures.[4] Briefly, polyoxymethylene window chambers, fabricated in house, were implanted on male athymic BALB/c nude mice (Taconic, Ry, Denmark) with 23-25 g BW. To generate tumor vasculature, 24 h after chamber implantation 5 × 10^6 MDA-MB-231 cells were implanted in the chamber center. The surgical procedures were performed under sterile conditions, and animals were allowed a standard rodent chow and water (67.5 mg l^{-1} enrofloxin (Baytril), 2% sucrose) *ad libitum*. Experiments began 12-16 days later. For imaging experiments, mice were anesthetized through subcutaneous injections of 12 mg kg^{-1} midazolam:fentanyl: Haldol:water (3:3:2:4), then cannulated in the tail vein, and fixed on a heated microscopy stage. The vasculature was imaged intravitally with a Leica SP8 confocal laser scanning microscope (CLSM; Leica, Wetzlar, Germany) using an HC/PL/APO 10x air objective with 0.4 numerical aperture.

For the intravital microscopy experiment, we used the FRET pair Cy3.5/Cy5 instead of Cy5.5/Cy7, because Cy7's emission wavelength is beyond the detection limit of the CLSM at our disposal. 8 window chamber mice were injected with NPs (n=1 for Cy3.5-NP control, n=2 for Cy3.5-NP:Cy5-X [X= OLA and PLGA2k], n=3 for Cy3.5-NP:Cy5-C12 at a dose of 1.5 g kg^{-1} BW). Directly after injection, we adjusted focus to the tumor vasculature that was subsequently imaged. 4 mice (n=1 for Cy3.5-NP or Cy3.5-NP:Cy5-X [X= C12, OLA and PLGA2k]) underwent 45 min of sequential spectral imaging at a temporal resolution of 12 s and spatial resolution of 2.27 μm^2

with the filter sets: λ_{Exc} = 570 nm, λ_{Em}=591±10, 602±10, 613±10, 624±10, 635±10, 646±10, 657±10, 668±10, 679±10, 690±10, and 701±10 nm. The other 4 mice (n=1 for Cy3.5-NP:Cy5-X [OLA and PLGA2k] and n=2 for Cy3.5-NP:Cy5-12) were subjected to 60 min of high spatial resolution (1.13 μm^2) sequential imaging at a temporal resolution of 3 min. Three optical channels were recorded: Cy3.5 (λ_{Exc} = 570 nm, λ_{Emi}=585-620 nm), FRET (λ_{Exc} = 570 nm, λ_{Emi}=655-730 nm), and Cy5 (λ_{Exc} = 633 nm, λ_{Emi}=655-730 nm). The recorded images were analyzed using Fiji/ImageJ2 software. The window chamber experiments were approved by the institutional ethics committee and were in accordance with the national (Norway) and institutional (NTNU) guidelines.

Pharmocokinetics and biodistribution of Cy5.5-NP:Cy7-X (X= CA, OLA, PLGA2k) nanoparticles

To measure the circulation half-life of the Cy5.5-NP:Cy7-X, nude mice without tumors (n=3) were injected with the nanoparticle solution via the tail vein at a dose of 1.5 g/kg. Blood samples were collected at selected post-injection time points (t=10 min, 30 min 1, 2, 4, 8, and 24 hour), weighted, and diluted in 50 μl of PBS solution containing 100mM EDTA. The blood samples were centrifuged at 10k rpm for 10 min and the plasma was collected. To extract the Cy5.5-PLGA and Cy7-X, 50 μl of the plasma was added to 950 μl acetonitrile and incubated overnight in darkness at 4 °C and then centrifuged at 10k rpm for 10 min to remove the insoluble part.

To quantify Cy5.5-NP:Cy7-X in tumor and organ tissue, nude mice bearing tumors (n=3) were injected with the nanoparticle solution via the tail vein at a dose of 1.5 g kg^{-1}. Tissue collection and subsequent extraction of Cy5.5-PLGA and Cy7 were similar to the procedure described for doxorubicin tissue distribution, except that acetonitrile was used for the extraction instead of acidic isopropanol.

A standard curve was prepared by making solutions of Cy5.5-CA or Cy7-CA in acetonitrile with concentrations from 0.01 ug ml^{-1} to 20 ug ml^{-1}. 200 µl of the extractions from plasma, tissues, or standard solution was loaded in a 96-well plate, and the fluorescence at both the Cy5.5 Cy7 channels was measured using a microplate reader. Auto-florescence from the samples was corrected by measuring the background fluorescence of the plasma or tissue extractions from the control mice (injected with PBS). The Mann-Whitney test was performed on the Cy7 concentration in tissue from different groups, and two-tailed *p* values were obtained using GraphPad Prism version 6.0 (GraphPad Software, La Jolla, CA).

Synthesizing Dox-X (X=C4, C18, PLA2k) parent drugs

The derivatives of doxorubicin's parent drug were synthesized by conjugating an aldoxorubicin (INNO-206; CAS#: 480998-12-7) (Medkoo Bioscience, Chapel Hill, NC) and a commercial available thiol ligand. The thiols used for synthesis were 1-butanethiol (99%; Sigma-Aldrich) for Dox-C4, 1-octadecanethiol (98%; Sigma-Aldrich) for Dox-C18, and poly(L-lactide) thiol terminated (Mn 2,500, PDI ≤1.3 from the manufacture; Sigma-Aldrich) for Dox-PLA2k. The reaction was generated by

dissolving aldoxorubicin and a thiol in anhydrous N,N-dimethylformamide (DMF; 99.8%; Sigma-Aldrich) in 1:1.2 molar ratio and stirring overnight under N_2 atmosphere. The reaction was monitored by TLC (eluted with chloroform:methanol, 4:1, v/v). After the reaction, a high-vacuum pumping removed the DMF. For Dox-C4 and Dox-C18, the crude product was washed with acetonitrile, and the undissolved part was collected through centrifugation. The sediment was redispersed in methanol and kept refrigerated for further use. For Dox-PLA2k, the crude product was washed twice before dissolving in acetonitrile and then precipitating down by adding methanol. Unreacted aldoxorubicin remained in the supernatant and was removed while discarding the supernatant. The final product was collected and redispersed in acetonitrile.

Synthesizing NP:Dox-X (X=C4, C18, PLA2k) nanoparticles

Dox-X loaded nanoparticle NP:Dox-X were synthesized through a nanoprecipitation method similar to the synthesis of Cy5.5-NP:Cy7-X. In a typical synthesis, 20 mg PLGA-PEG, 4.2 mg PLGA, and Dox-X were dissolved in 2.5 ml acetonitrile, and then the mixture was dripped into 20 ml PBS under vigorous stirring at room temperature. After continuous stirring for 1h, the product nanoparticles were first washed through centrifugation at 18.0 g for 10 min to remove possible aggregates, then washed at least 3 times, and finally concentrated in sterilized PBS with centrifugal concentrators (MWCO 100k) or Vivaflow 50 crossflow cassette (MWCO 100k; Sartorius AG, Göttingen, Germany).

To determine the doxorubicin concentration of the NP solution, a standard curve of doxorubicin with concentrations from 0.1 μg ml^{-1} to 50 μg ml^{-1} in DMSO was prepared. An aliquot of 10 μl of NP solution was added into 990 μl of DMSO and mixed by vortex. Triplicate of 200 μl of this solution and standard solutions were added into 96-well plates, and the absorbance at 500 nm was measured using a microplate reader.

Cellular uptake of NP:Dox-X by fluorescence microscopy

One day before the experiment, 4T1 cells were seeded on an 8-well Nunc Lab-Tek II chamber slide (Thermo Fisher Scientific, Waltham, MA, USA), at a density of 2 x 10^4 cells per well, and allowed to attach overnight. Cells were incubated at 37 °C in media containing different NP:Dox-X (X=C4, C18, PLA2k) nanoparticles at a final Dox equiv. concentration of 25 μM, Cy3.5-NP carrier control, and blank control. At 5 min, 30 min, 2h, or 24h after incubation, the solution mixture was removed and washed 3 times with fresh PBS. The cells were fixed in 4% paraformaldehyde, washed again with PBS 3 times, embedded in Vectashield antifade mounting medium with DAPI (Vector Lab, Burlingame, CA), and sealed under cover slides. The slides were imaged using Zeiss AxioImager 2 fluorescence microscope (Carl Zeiss AG, Jena, Germany) with a x40 oil-immersion objective. The filter sets were DAPI (λ_{Exc} = 357±22 nm, λ_{Emi}=447±30 nm) and Dox (λ_{Exc} = 585±15 nm, λ_{Emi}=624±10 nm).

Cytotoxicity of NP:Dox-X (X=C4, C18, PLA2k) nanoparticles

The NP:Dox-X therapeutic nanoparticles' cytotoxicity was determined in vitro using a MTT colorimetric assay. In 96-well plates with clear bottom, $5x10^3$ 4T1 cells were seeded in each well and allowed to attach overnight. Cells were then incubated with 175 µl of cell media and 25 µl of NP:Dox-C4, NP:Dox-C18, NP:Dox-PLA2k, or PBS control was added into each well (n=6), reaching final doxorubicin equivalent concentration of 25, 12.5, 0.625, 0.125, or 0 µM. After 2 days of incubation at 37 °C, the cell medium containing nanoparticles was removed, and 100 µl of fresh culture medium containing 10 µl of the 12 mM 3-(4,5-dimethylthiazol-2-yl)-2,5-diphenyltetrazolium bromide (MTT) stock solution (Thermo Fisher Scientific) was added to each well and the cells were incubated at 37 °C for one hour. For negative control, we added the same MTT solution to 100 µl medium alone. Finally, the incubation mixture was removed and 100 µl of DMSO was added to each well yielding a homogenized solution. The absorbance at 500 nm was measured using a microplate reader. The cell viability percentage was calculated by subtracting the absorbance value of negative control from that of each sample well and then normalized to 100% viability (i.e. the PBS-incubated cell).

Supplementary References:

1. Fedors, R.F. A method for estimating both the solubility parameters and molar volumes of liquids. *Polym. Eng. Sci.* **14**, 147-154 (1974).
2. van Krevelen, D.W. & te Nijenhuis, K. Properties of Polymers: Their Correlation with Chemical Structure; their Numerical Estimation and Prediction from Additive Group Contributions. (Elsevier Science, 2009).
3. Kim, Y. et al. Probing nanoparticle translocation across the permeable endothelium in experimental atherosclerosis. *Proc. Natl. Acad. Sci. USA* **111**, 1078-1083 (2014).
4. Hak, S. et al. The Effect of Nanoparticle Polyethylene Glycol Surface Density on Ligand-Directed Tumor Targeting Studied in Vivo by Dual Modality Imaging. *ACS Nano* **6**, 5648-5658 (2012).
